# Eumetazoan Cryptochrome Phylogeny and Evolution

**DOI:** 10.1093/gbe/evv010

**Published:** 2015-01-18

**Authors:** Marion F. Haug, Matthias Gesemann, Viktor Lazović, Stephan C.F. Neuhauss

**Affiliations:** Institute of Molecular Life Sciences, Neuroscience Center Zurich and Center for Integrative Human Physiology, University of Zurich, Switzerland

**Keywords:** cryptochrome, zebrafish, evolution, phylogeny, circadian

## Abstract

Cryptochromes (Crys) are light sensing receptors that are present in all eukaryotes. They mainly absorb light in the UV/blue spectrum. The extant Crys consist of two subfamilies, which are descendants of photolyases but are now involved in the regulation of circadian rhythms. So far, knowledge about the evolution, phylogeny, and expression of *cry* genes is still scarce. The inclusion of *cry* sequences from a wide range of bilaterian species allowed us to analyze their phylogeny in detail, identifying six major Cry subgroups. Selective gene inactivations and stabilizations in multiple chordate as well as arthropod lineages suggest several sub- and/or neofunctionalization events. An expression study performed in zebrafish, the model organism harboring the largest amount of *crys*, showed indeed only partially overlapping expression of paralogous mRNA, supporting gene sub- and/or neofunctionalization. Moreover, the daily *cry* expression in the adult zebrafish retina indicated varying oscillation patterns in different cell types. Our extensive phylogenetic analysis provides for the first time an overview of *cry* evolutionary history. Although several, especially parasitic or blind species, have lost all *cry* genes, crustaceans have retained up to three *crys*, teleosts possess up to seven, and tetrapods up to four *crys*. The broad and cyclic expression pattern of all *cry* transcripts in zebrafish retinal layers implies an involvement in retinal circadian processes and supports the hypothesis of several autonomous circadian clocks present in the vertebrate retina.

## Introduction

Cryptochromes (Crys) were first described as “cryptic” blue-light photoreceptors in plants that control aspects of growth and development (reviewed in Lin [2000]). Only in the 1990s, the first *cryptochrome* gene was cloned and identified as a UV- and blue-light absorbing flavoprotein, closely related to DNA photolyases (Ahmad and Cashmore 1993). Both, cryptochromes and photolyases, share a conserved photolyase-related region that binds two chromophores: FAD (flavin adenine dinucleotide) and in the case of cryptochromes predominantly MTHF (methenyltetrahydrofolate) (reviewed in Sancar [2003] and Chaves et al. [2011]). Although in photolyases light absorption enables the repair of UV-induced DNA damage, most Crys have partially or completely lost this function but gained novel roles in signaling (Malhotra et al. 1995; reviewed in Sancar [2003] and Chaves et al. [2011]).

Crys are widely distributed in pro- and eukaryotes (with the exception of archaea), where they are involved in a variety of light responses, most prominently in circadian activity regulation (reviewed in Chaves et al. [2011]). Animal-type cryptochromes are divided into two functional groups. Type I cryptochromes, also known as *Drosophila*-type cryptochromes, are directly light-sensitive and act as the circadian photoreceptor, as had been described in detail in *Drosophila* (Emery et al. 1998; Stanewsky et al*.* 1998). On the other hand, no light-dependent function has been described for type II cryptochromes (vertebrate-type cryptochromes) which mostly regulate the transcription of clock genes in the negative limb of the feedback loop of the circadian clock (Kume et al. 1999; Shearman et al. 2000). In some species such as *Arabidopsis*, cyanobacteria, or zebrafish, another type of cryptochrome, CryDASH, has been described (Hitomi et al. 2000; Brudler et al. 2003; Daiyasu et al. 2004). As they have retained the ability of photorepair, they are described as an intermediate form between photolyases and Cryptochromes (reviewed in Chaves et al. [2011]). CryDASHs build a monophyletic group in the Cryptochrome/Photolyase family and are closer related to plant-type Cryptochromes and CPD (cyclobutane pyrimidine dimer) photolyases than to animal-type Cryptochromes (Oliveri et al. 2014). As our study focuses on the phylogeny of animal-type Cryptochromes that can be found in animals as well as plants, the functionally and phylogenetically only distantly related CryDASHs were not included. For the remainder of the article, we will use the term Cryptochrome as a synonym for these animal-type Cryptochromes.

The cryptochrome content in vertebrate genomes was altered during evolution by several rounds of whole-genome duplication (WGD) events. There is evidence for the occurrence of two such large-scale genomic events in vertebrates and an additional genome duplication event that happened about 350 Ma at the base of the teleost lineage (Amores et al. 1998; Vandepoele et al. 2004; Glasauer and Neuhauss 2014). Such events likely have built the basis of species radiation (Aury et al*.* 2006; reviewed in Ohno [1999] and Volff [2005]). Despite two WGD events mammals only possess the two type II cryptochromes Cry1 and Cry2 (Hsu et al. 1996). However, some species such as zebrafish (Kobayashi et al. 2000) or chicken (Ozturk et al. 2009) maintain a higher number of *cryptochromes* within their genome. Among all animals, zebrafish and their close relatives, the cavefish, harbor the largest reported Cry family with seven members and a still active photolyase in their genome (Kobayashi et al. 2000; Daiyasu et al. 2004; Tamai et al. 2004; Cavallari et al. 2011).

In contrast to vertebrates, invertebrate evolution lacks such additional rounds of WGDs at the basis of their lineage and no more than three *cryptochromes* have been described in investigated invertebrate genomes (Yuan et al. 2007; Bertossa et al. 2014). Although some studies provide a detailed analysis of the cryptochrome gene family (Kobayashi et al. 2000; Lin and Todo 2005; Yuan et al. 2007; Kubo et al. 2010; Bertossa et al. 2014; Oliveri et al. 2014), they only include few species and a broad overview of the Cryptochrome phylogeny of invertebrate and vertebrate species is missing.

Here, we investigated the evolutionary relationships of Crys in a large range of eumetazoan species thus providing a complete overview of cryptochrome phylogeny. We found that during evolution, WGD events followed by non-, sub-, and/or neofunctionalization led to the huge variability in *cry* gene number in different species. As zebrafish possess at least one representative of each Cryptochrome and as this organism, due to its genomic and genetic resources, emerges as a useful model to study the fate of genes throughout evolution, we analyzed the expression of each *cry* transcript in larval zebrafish. The study was completed with an expression analysis in adult zebrafish retinae to conclude about possible nonvisual regulatory functions of each Cryptochrome.

## Materials and Methods

### Fish Maintenance and Breeding

Adult fish were kept under standard conditions at a 14 h/10 h light/dark cycle at 28 °C. For this study, only fish of the wild-type strain “Tü” were used (Haffter et al. 1996). Embryos were raised at 28 °C in E3 medium (5 mM NaCl, 0.17 mM KCl, 0.33 mM CaCl_2_, and 0.33 mM MgSO_4_) and staged according to development in days postfertilization (dpf). All animal experiments were performed in accordance with the ARVO Statement for the Use of Animals in Ophthalmic and Vision Research and were approved by the local authorities (Veterinäramt Zürich TV4206).

### Annotation of *cryptochrome* Sequences

As gene predictions produced by automated processes have been shown to contain numerous errors, *cry* cDNA sequences used in this study were manually annotated. Sequences were identified and annotated using combined information from expressed sequence tags and genome databases (GeneBank, http://www.ncbi.nlm.nih.gov, last accessed February 2, 2015; Ensembl, http://www.ensembl.org/index.html, last accessed February 2, 2015). Human and mouse sequences were used as initial query (for more details on sequence annotation, see Gesemann et al. 2010). Exon sizes as well as putative cDNA length of cryptochromes from related species were further used as reference. The sequences of all eight zebrafish cryptochromes have been previously determined (Kobayashi et al. 2000) and were confirmed in our lab by reannotations, cloning from zebrafish cDNA and subsequent sequencing.

### Phylogenetic Tree Analysis

Phylogenetic analysis was performed on the Phylogeny.fr platform (http://www.phylogeny.fr/, last accessed February 2, 2015) (Dereeper et al. 2008). Chryptochrome cDNA sequences were translated into amino acid sequences and subsequently aligned using MUSCLE v3.7 (Edgar 2004) configured for highest accuracy (MUSCLE with default settings). Length of input sequences varied between 501 and 961 amino acids. After alignment, ambiguous regions (i.e., containing gaps and/or being poorly aligned) were removed with Gblocks v0.91b (Castresana 2000) using the following parameters: The minimal length of a block after gap clearing was set to 10 and no gap positions in the final alignment were allowed. Alignments with continuous nonconserved positions larger than 8 were rejected and at least 85% of the sequences had to be present at gap flanking positions. Following curation 292 amino acids were used for further analysis. Corresponding nucleotide sequences of the curated amino acid sequences were used for phylogenetic trees reconstruction using the maximum-likelihood method implemented in the PhyML program v3.0 (Guindon and Gascuel 2003). The gamma shape parameter was estimated directly from the data. Branch reliability was assessed by the approximate likelihood-ratio test (aLRT, SH-like) (Anisimova and Gascuel 2006). Graphical representations of the phylogenetic trees were obtained using TreeDyn v198.3 and edited in Coral draw (Coral Corporation).

Accession numbers and information on the used sequences are listed in supplementary table S1, Supplementary Material online.

### Synteny Analysis

An initial rough synteny analysis was done using the synteny database (http://syntenydb.uoregon.edu/synteny_db/, last accessed February 2, 2015) (Catchen et al. 2009). Synteny hits in the output files were further subjected to a microsynteny analyses, were paralogous/orthologous genes of human or zebrafish, were used as initial queries for a tBLASTx search against the zebrafish or human database (ncbi nr/nt database). The hit with the highest conservation (length and identity) was used in a reciprocal tBLASTx search against the corresponding database and only genes identifying the initially used query are counted as positive matches.

### Cloning of Zebrafish *cryptochromes*

Sequences of the previously described zebrafish *crys* were downloaded from zfin (http://zfin.org, last accessed February 2, 2015) and various primers for PCR amplification were ordered (Sigma-Aldrich, Buchs SG, Switzerland). Amplified fragments were sequenced and were purified using the NucleoSpin Extract II kit (Macherey-Nagel, Oensingen, Switzerland) and subsequently cloned into a pCRII vector (pCRII TOPO TA-cloning kit; Invitrogen, Life Technologies, Zug, Switzerland). An amount of 6 µl of ligated plasmid DNA were added to 50 µl of bacterial suspension (OneShot TOP10; Invitrogen), left on ice for 20 min before being incubated for 40 s at 42 °C and again placed on ice for 2 min. An amount of 500 µl prewarmed, sterile 25% lysogeny broth (LB)-medium (Luria Broth Base; Invitrogen) was added and the cell suspension was incubated for 1 h at 37 °C in a gently shaking incubator. Afterwards, the cells were collected by centrifugation (8,000 rpm for 3 min). The supernatant was discarded, the cells resuspended and plated onto prewarmed agar plates containing ampicillin (0.1 mg/ml), Isopropyl β-D-1-thiogalactopyranoside (IPTG); 0.71 µg/ml; Roche, Basel, Switzerland), and X-Gal (5-bromo-4-chloro-3-indolyl-β-D-galactopyranoside; 48 µg/ml; Roche). The plates were incubated over night (ON) at 37 °C. The next day, colonies were picked using a sterile pipette tip, transferred to a culture tube containing 5 ml of 25% LB-medium with ampicillin (0.1 mg/ml), and incubated ON in a shaking incubator at 37 °C. Plasmid DNA was isolated and purified with the NucleoSpin Plasmid kit (Macherey-Nagel), concentration was measured using a NanoDrop (ND-1000; Witec AG, Litau, Switzerland). Subsequently, plasmids were sequenced in house.

### In Situ Hybridization

The primers used for probe preparation are listed in table 1. Plasmids were linearized for T7 and Sp6, in vitro transcribed, and purified on a column (Macherey-Nagel). The probes were DIG-labeled using a kit (DIG-RNA labeling kit; Roche). Transcripts were hydrolyzed to obtain fragments of approximately 300–500 nucleotides of length. As working probes a mixture of nonhydrolyzed (2 ng/µl) and hydrolyzed (1 ng/µl) probe was used.

**Table 1:** Primer Pairs Used for Riboprobe Preparation and qRT-PCR Analysis

Gene	ISH		qRT-PCR	
	5′–3′		5′–3′	
*cry1a*	F:	GAGAGCAGTTTCTTTTTTGG	F:	CAGGCGTGGAGGTGATAG
	R:	AAGCCTCTGGGTTTTTATC	R:	TGGAAGCGCTTATACGTG
*cry1b*	F:	GGATCTCCCACACACTCTATG	F:	ACACCGGTCAGTGATGATC
	R:	CACGTGTGAGGAAGCAAG	R:	TGGACAGTCCCTCTGTTTC
*cry3a*	F:	GGACTGACATAACGTTAAAAG	F:	GCTGTTGCATGTTTCCTC
	R:	CAGTCTGCATCCAATAGAAG	R:	CAGTCTGCATCCAAATAGAAG
*cry3b*	F:	TCTGCATTATTGACAGCTTG	F:	CCGGTGGAGAATCAGAAG
	R:	CCCCACAGGACAGTAACAG	R:	TTCGGTCGCTCAAAGTTC
*cry2*	F:	ATTTCCTTGGAACTTTTACG	F:	GACATGCAGTAGCCTGTTTC
	R:	CGCATCCAACAGCAACTC	R:	CGCATCCAACAGCAACTC
*cry4*	F:	CTACGCACAGTCGAAGAAC	F:	CGAACCTTCTACCACAGACTC
	R:	CTTCTGGGTCGTAAAACATTC	R:	AGCGACGGTGTAGAAGAAC
*cry5/6-4phr*	F:	CTGGGTGTGCAAGTTTGAG	F:	ACCCATTCCTGCTCCAAC
	R:	ATGGATGGACTCGCTTTG	R:	CAGTCCAAGATCCTCAAGAG
*Rpl-13a*			F:	GGACTGTAAGAGGTATGCTTC
			R:	GATGCCATCAAACACCTTC
*EF1α*			F:	GAGGCCAGCTCAAACATG
			R:	TCAAGGGCATCAAGAAGAG
*prkcα*			F:	GGACTCATACACCAAGGAATG
			R:	GCTTGGCACATTCATCAC

Embryos used for in situ hybridization (ISH) were treated with 0.2 mM PTU (1-phenyl-2-thiourea; Sigma-Aldrich) to prevent melanization of skin melanocytes and the retinal pigment epithelium. At the fifth dpf, embryos were fixed in 4% paraformaldehyde (PFA; Sigma) in phosphate-buffered saline (PBS), pH 7.4 at the appropriate time point (according to the highest expression level found in adult retinae by qRT-PCR analysis: 7 am for *cry1b* and *cry2*, 11 am for *cry1a* and *cry5/6-4phr*, 11 pm for both *cry3* paralogs and *cry4*) ON at 4 °C. The next day, the embryos were washed twice in PBS containing 1% Tween (PBT), dehydrated in a graded series of PBT:MeOH mixtures (3:1; 1:1; 1:3), and stored in 100% MeOH at −20 °C until further use. Adult zebrafish were euthanized with tricaine (MS-222; Sigma-Aldrich) in iced water at the appropriate time point (7 am, 11 am, 7 pm, 11 pm). Eyecups were removed and fixed ON at 4 °C in 4% PFA in PBS, pH 7.4. ISH and imaging of whole mount larvae and of sections were performed as previously described (Haug et al. 2013). Images were processed and arranged with Adobe Photoshop and Illustrator CS5.

### Quantitative Real-Time PCR

After removing the eyecups of the fish at the appropriate time point (ZTs 3, 7, 11, 15, 19, 23), the tissue was collected in RLT buffer (Qiagen, Hombrechtikon, Switzerland), pounded with a pistil and homogenized with a sonificator (Sonopuls HD2070; Bandelin Electronic, Berlin, Germany). Fish were euthanized in darkness and eyecups were removed under dim red light when the tissue was collected during the dark period. RNA was extracted with the NucleoSpin RNA II Kit (Macherey-Nagel) and RNA concentration was measured with a NanoDrop (ND-1000; Witec AG, Litau, Switzerland). Reverse transcription was performed with 400 ng RNA and the Superscript II kit (Invitrogen, Life Technologies, Zug, Switzerland). Quantitative real time polymerase chain reaction (qRT-PCR) was performed in a transcriptor (Applied Biosystems Prism SDS 7900HT; Life Technologies) using the MESA Green Kit (Eurogentec, Seraing, Belgium). Primer pairs (Sigma-Aldrich) used for qRT-PCR were specifically designed to incorporate an intron to avoid unspecific amplification of genomic DNA (see table 1). As a reference, the genes *Rpl-13α* (ribosomal protein L-13a), *EF1α* (elongation factor 1 alpha), and *prkcα* (protein kinase C alpha) were selected (Tang et al. 2007). The highest expression of each gene was given 1 and lower expression was set relative to this total. The values are means ± standard deviations and were averaged from three independent samples. Analysis was completed in Microsoft Excel and data were analyzed by a one-way analysis of variance (ANOVA) (*P* < 0.05) and a Tukey’s Multiple Comparsion Test in GraphPad Prism 5. Finally, graphs were arranged in Microsoft Excel and Adobe Illustrator CS5.

## Results

### Eumetazoan Cryptochrome Phylogeny and Nomenclature

**Fig. 1.—:**
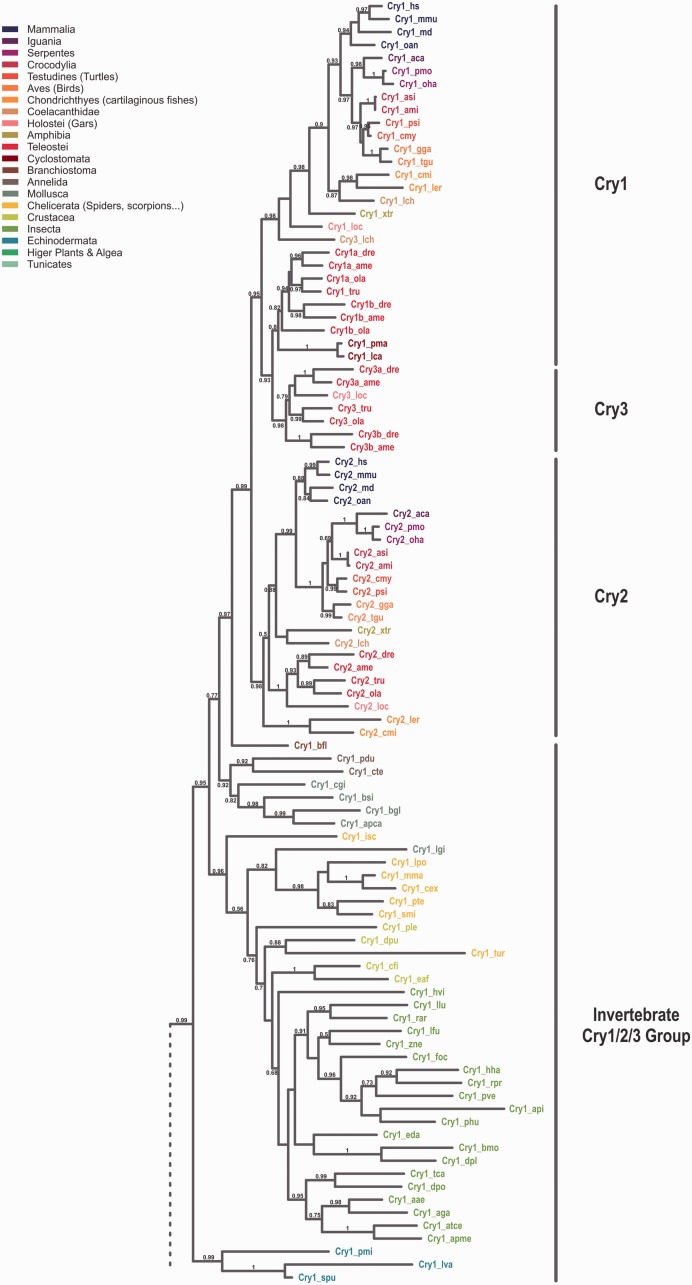

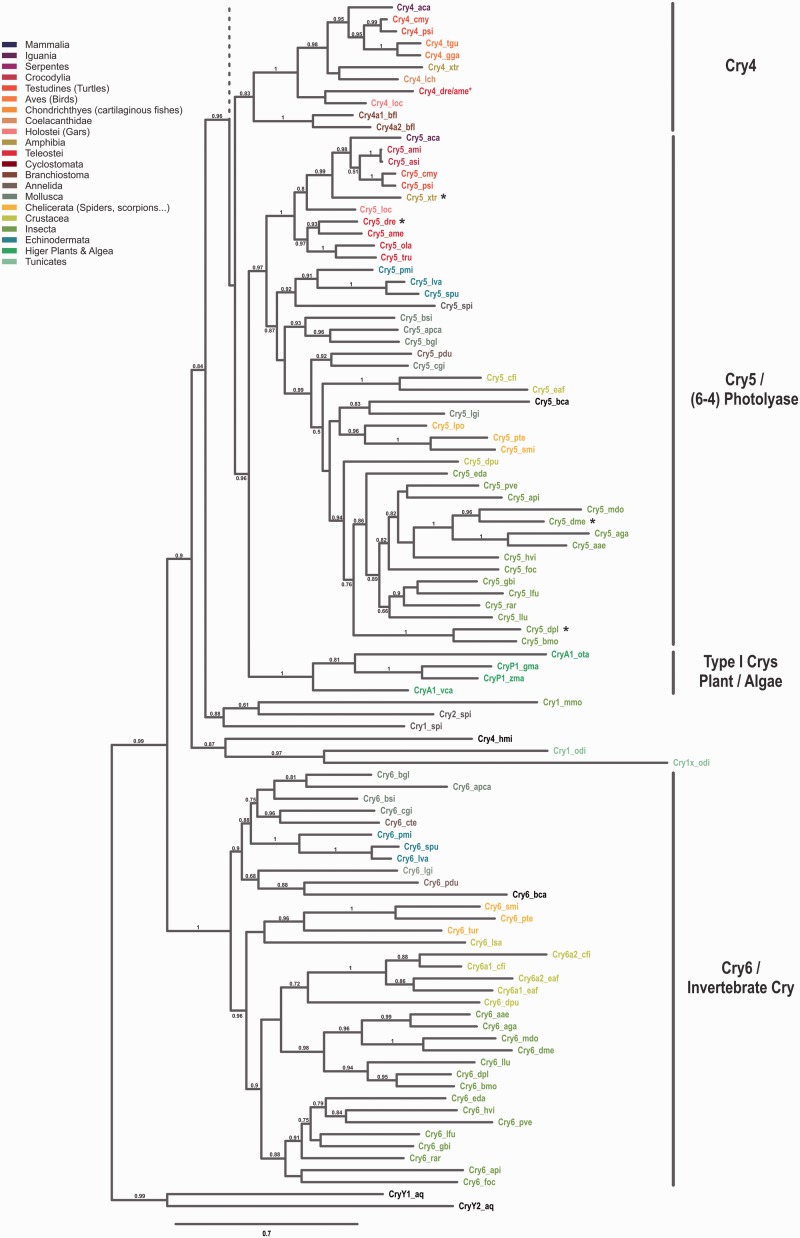
Phylogeny of the Cryptochrome family. Maximum-likelihood analyses of cryptochromes from bilaterian species reveal six major subgroups. The phylogenetic tree was built using the corresponding 876 nucleotides obtained from a block of 292 conserved amino acids. Conserved amino acids were determined by the program Gblocks on a sequence alignment done by MUSCLE. Wherever available at least two species of neighboring classes/orders with distinguishable cryptochrome content are included. The different classes/orders/suborders are highlighted at the side and the cryptochomes of the species used are color coded accordingly. Full names for the species used can be found in the supplementary table S1, Supplementary Material online. Bootstrap values above 50% (0.5) are shown. The scale bar shows the percentage (0.7 equals 70%) of nucleotide substitutions required to generate the corresponding tree. (*A*) Grouping of cryptochromes in subgroups 1–3. Note that members of the Cry3 subfamily are exclusively found in ray finned fish and that invertebrates in general possess only one representative Cry1/2/3 sequence. Interestingly, the Cry1 ortholog found in jawless vertebrates (represented by lampreys) can be found in a clade with teleost Cry1/3 genes. (*B*) Grouping of cryptochromes in subgroups 4–6. Although invertebrates lack a Cry4 homolog, chordates have lost a corresponding Cry6 variant. Cryptochromes in the Cry5/6-4 PhR clade with proven photolyase activity are highlighted by black asterisks. Note that the included cryptochromes from plants and algae form a separate branch that has not been assigned to any of the six cryptochrome subgroups. The red asterisk depicted after the zebrafish (dr)/cavefish (ame) Cry4 indicates that, due to the incomplete, but clearly present, cavefish Cry4 sequence only the zebrafish variant was used for phylogenetic reconstruction. We routed the tree with the two distantly related cryptochromes from the sponge *Amphimedon queenslandia* as outgroups.

To study the evolutionary relationship among Cryptochromes, we performed a phylogenetic analysis using sequences of more than 100 eumetazoan species ranging from cnidaria to mammals (fig. 1). To simplify matters, in this publication we only consider the Cryptochromes after the CryDASH/Cryptochrome split, although we are aware of the fact that CryDASHs may also be considered as part of the Cryptochrome gene family.

**Fig. 2.—:**
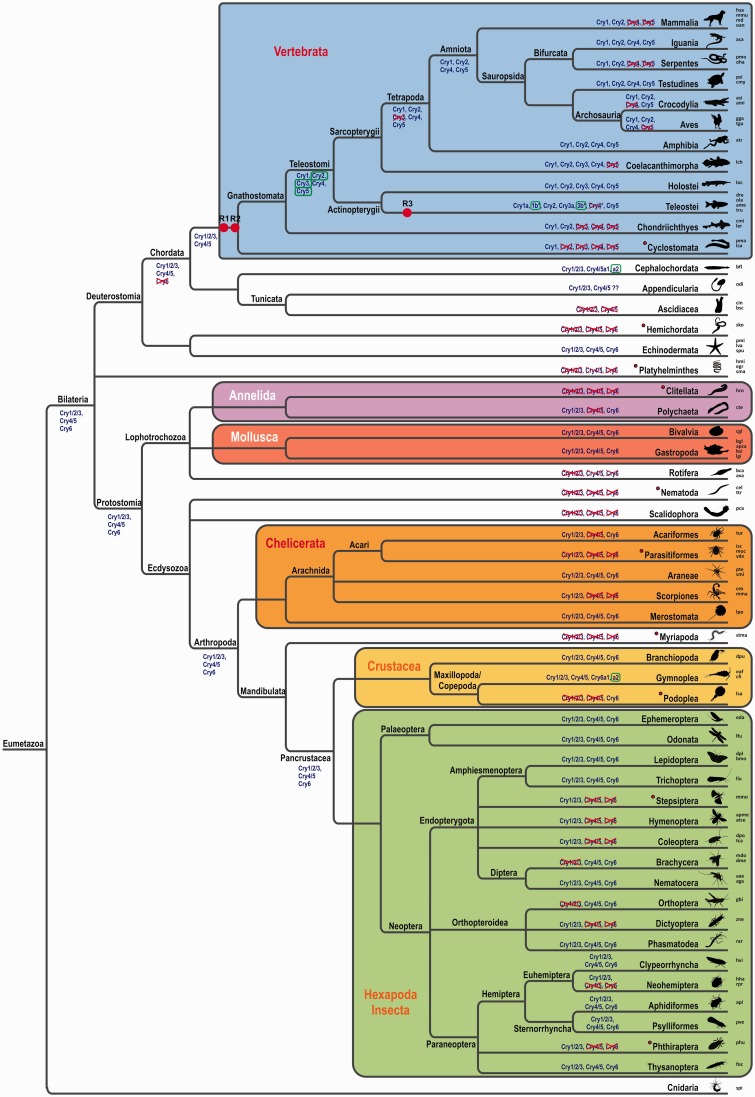
Evolution of the *cryptochrome* genes. The phylogenetic relation of major subtypes of bilaterian species is represented in the depicted tree. Order, classes, and phylum names at branch points are indicated. Note that the branch lengths are NOT scaled with time. The major phyla and subphyla are highlighted by colored boxes. A representative image of one species for each order/class/phylum is given for better orientation. The abbreviation of the species used for phylogenetic reconstruction is shown at the right-hand side and corresponding species names are summarized in supplementary table S1, Supplementary Material online. Verified WGD events are indicated by red dots and labeled as R1, R2, and R3. Putative *cryptochrome* contents at extrapolated ancesteral stages are given at selected branch points and *cryptochrome* gains (green boxes) and losses (red crosses) are indicated. Note that parasitic or light independent species (red filled cycles) often lack *cryptochrome* genes or only have one variant left. Overall, multiple independent gene losses can be seen in isolated orders or classes.

We found a huge variability in *cryptochrome* number and identity throughout the different animal phyla. Although mammalian genomes are limited to two *cryptochrome* genes, sauropsida genomes harbor two to four *crys*. Four *cryptochromes* can be found in amphibia and coelacants, whereas the genomic *cry* content in ray-finned fishes reaches as much as seven members, mainly due to the teleost-specific WGD (TGD) (figs. 1*A*, *B* and 2). Prior to the two vertebrate specific WGDs we found species with no *cryptochrome* (*Ciona intestinalis*, leech, some mites), one (scorpions and some insects), two (some mites and insects), or three *crys* (cephalochordates, echinodermates, mollusks, spiders, and some insects). In all these lineages, different *cryptochromes* were lost independently. Interestingly, all insects possess at least one *cryptochrome* and more than half of the insect lineages kept the three ancestral *cry* genes although in other invertebrate lineages the loss of *cryptochromes* occurred much more frequently. Among the few invertebrate species besides the insects that still harbor all three *cry* genes are the echinodermata, mollusks, aranea, merostomata, and gymnoplea. The latter (e.g., the zooplankton *Calanus finmarchicus*) even possess four *cryptochromes*, most likely due to a tandem duplication event (fig. 2).

**Table 2:** Overview of *cry* Names

New Name	*Mus musculus*	*Gallus gallus*	*Xenopus tropicalis/laevis*	*Danio rerio*
*cry1 (a/b)*	*cry1*	*cry1*	*cry1*	*cry1a, cry1b*
*cry3 (a/b)*	*/*	*/*	*/*	*cry2a, cry2b*
*cry2*	*cry2*	*cry2*	***cry2/cry2a, cry2b*^a^**	***cry3***
*cry4 (double function cry)*	*/*	***cry4***	***cry1, cry4***	***cry4***
*cry5 / (6-4) phr*	*/*	*/*	***6-4 photolyase***	***6-4 photolyase***
*cry6*	*/*	*/*	*/*	*/*

	*Danaus plexippus*	*Drosophila melanogaster*	*Apis mellifera*	
*cry1 (cry1/2/3)*	*cry2*	*/*	*cry2*	
*cry5 (cry4/5)*	***phr6-4***	***dm64, phr6-4***	*/*	
*cry6*	***cry, cry1***	***dmcry, dcry, cry1***	*/*	

Note.—Type I or Drosophila-type cryptochromes are given in bold.

Our extensive phylogenetic analyses led us to propose the renaming of certain Crys according to their arrangement in monophyletic groups (see table 2 for an overview of the new and old names for selected species). We and others found that the zebrafish and other teleost genomes harbor three to four Cry1 orthologs. In zebrafish, the paralogs were initially named Cry1a and –b, and Cry2a and –b, indicating their close relationship (Kobayashi et al. 2000). However, the zebrafish Cry originally named Cryptochrome 3 clearly groups with the mammalian Cry2 forming a monophyletic clade. To stay consistent with the phylogenetic data, we propose to rename the teleost Cry3 to Cry2. This has the consequence that the initial Cry2s have to be renamed to Cry3. As the initially named Cry3 genes are only present in teleosts, the renaming will be limited to only a few database entries. Although this renaming has the consequence that teleost Cry1s and Cry2s are no longer the closest relatives, it has the clear advantage that no other Crys throughout the animal phylum have to be renamed. Cry4 and Cry5 named genes in the database could be maintained. Consistent with some database entries, the Cryptochrome photolyase or (6-4) PhR is now named Cry5 or Cry5/6-4PhR in case of proven photolyase activity. The last group of Cryptochromes, exclusively found in invertebrates, we now call Cry6. Figure 2 summarizes the phylogenetic tree thus providing an overview of the phylogenetic relation of all major subtypes of bilaterian species. Gain and loss of Crys in each lineage can clearly be identified which supports the understanding of each species’ Cry content.

**Table 3:** Overview of *cry* Expression in Zebrafish

Gene	Region														
	5dpf			Adult Retina											
	CNS	Retina	Body	ONL				INL				GCL			
				ZT3	ZT11	ZT15	ZT23	ZT3	ZT11	ZT15	ZT23	ZT3	ZT11	ZT15	ZT23
*cry1a*	Die ++ TeO ++ MO ++	INL (+) INLp (++) GCL (++)	−	+	±	−	+	++	+	±	++	+	+	+	+
*cry1b*	−	INLp (+) GCL (+)	−	m ± p +	m ± p +	m + p −	m ± p +	++	+	±	++	±	−	−	++
*cry3a*	S (++)	ONL (++) INL (+) INLp (+) GCL (+)	Int (++)	±	+	++	−	+ d −	++ d +	++ d ++	− d −	±	++	+	−
*cry3b*	TeO (++)	ONL (++) INL (+) INLp (+) GCL (+)	Int (++)	−	+	+	−	+ d −	++ d +	++ d ++	− d −	+	++	++	−
*cry2*	TeO ++ T ++ MO ++	ONL (++) INL (+) INLp (+) GCL (+)	−	m − p +	m − p −	m − p −	m ++ p −	++	±	±	++	++	−	−	+
*cry4*	TeO (++) overall broad	ONL (++) INL (+) INLp (+) GCL (+)	Int (++)	±	+	+	−	++	++	++	−	−	±	++	−
*Cry5/6-4phr*	TeO (++) overall broad	INLp (+) GCL (±)	−	+	−	−	−	+	−	−	±	±	−	−	−

Note.—“−,” no expression; +/–, very weak expression; +, medium expression; ++, strong expression. Expression intensity in specific areas was semiquantitatively evaluated by appearance of the intensity of blue staining. Die, diencephalon; INLd, distal INL; INLp, proximal INL; Int, intestine; MO, medulla oblongata; ONLm, medial ONL; ONLp, proximal INL; S, subpallium; T, tegmentum; TeO, optic tectum.

**Fig. 3.—:**
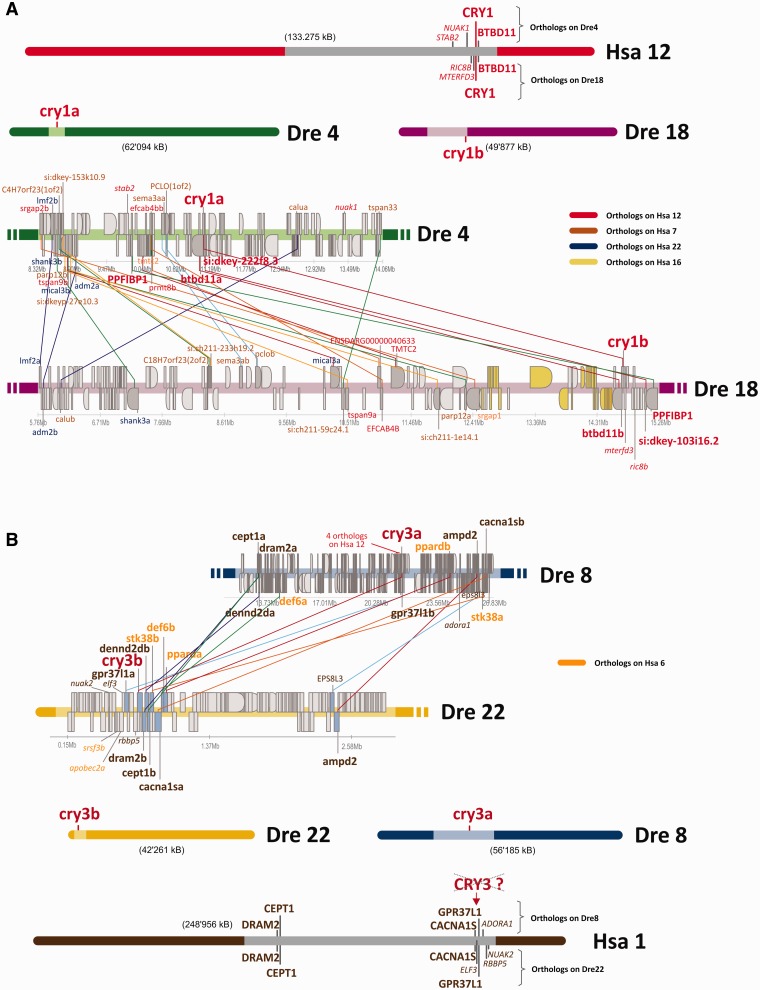
Synteny of the zebrafish *cry1/cry3* genes. Genomic regions around the zebrafish *cry1a/cry1b* (A) and *cry3a/cry3b* (B) paralogs are shown. The general location of *cry* genes on human and zebrafish chromosomes is depicted at the top (*A*) or bottom (*B*) of the figure. Scale ups of the light colored regions of the corresponding chromosome are used to depict the synteny. *cry* genes are highlighted in big, bold, red letters. Adjacent to the *cry1a* and the *cry1b* gene, three other paralogous gene pairs, namely *PPFIBP1*, *btbd11* and *si:dkey103i16.2/222f8.3* (bold, red), can be found on both zebrafish chromosomes 4 and 18. *btbd11* is also the direct neighbor of *cry1* on human chromosome 12, indicating conserved synteny. Note that additional zebrafish genes in the vicinity of *cry1a/cry1b* (e.g., *nuak1*, *stab2*, *ric8b*, *mterfd*; italic red) also have their orthologs on human chromosome 12 close to the *cry1* gene. Paralogous genes between zebrafish chromosomes are indicated by colored lines. Ortholog location of corresponding human genes is color coded as indicated at the right site of the figure. Zebrafish genes around the *cry1a/cry1b* genes have their orthologs mainly on human chromosomes 12, 7, and 22. In the case of *cry1b* an additional island of genes with orthologs on human chromosome 16 can be found. In contrast to *cry1a* and *cry1b*, genes flanking *cry3a* and *cry3b* have their orthologs mainly on human chromosomes 1 and 6, indicating that the origin of *cry1* and *cry3* genes is different and that a putative, now lost *cry3* might have been located on human chromosome 1. This is especially apparent in the situation of *cry3b*, where all zebrafish genes flanking *cry3b* have their orthologs on human chromosome 1 (brown coloring).

To ascertain that the zebrafish Cry1 and Cry3 paralogs originate from a WGD event, we investigated their genomic regions. All four zebrafish *cry1* orthologs are located on different chromosomes and within close proximity of either the *cry1* or the *cry3* regions we found other duplicated genes specific for each two paralogs (red lines, fig. 3). This conserved synteny indicates that they are ohnologs that originated from a WGD event. Moreover, synteny analysis between the human *cry1* and the zebrafish *cry1* and *cry3* paralogs clearly displays a conserved synteny of the human and the zebrafish *cry1s* (fig. 3). In the case of the *cry1* paralogs, the same genes are located in both genomic surroundings as were found in close proximity of the human *cry1* on chromosome 12 (e.g., btbd11 in both cases and either stab2 for *cry1a* or ric8b for *cry1b*).

The last common ancestor representing all Crys 1, 2, and 3 of vertebrates and invertebrates we called Cry1/2/3 to indicate its relationship. Subsequent WGD events (depicted as R1, R2, and R3 in fig. 2) led to the increase in Cry1/2/3 orthologs in the vertebrate lineage, whereas invertebrates still possess only one ortholog depicted as Cry1 in the phylogenetic tree.

### Expression of *cry* Transcripts in Larval Zebrafish

The zebrafish genome harbors at least one member of each original vertebrate *cryptochrome*. 

**Fig. 4.—:**
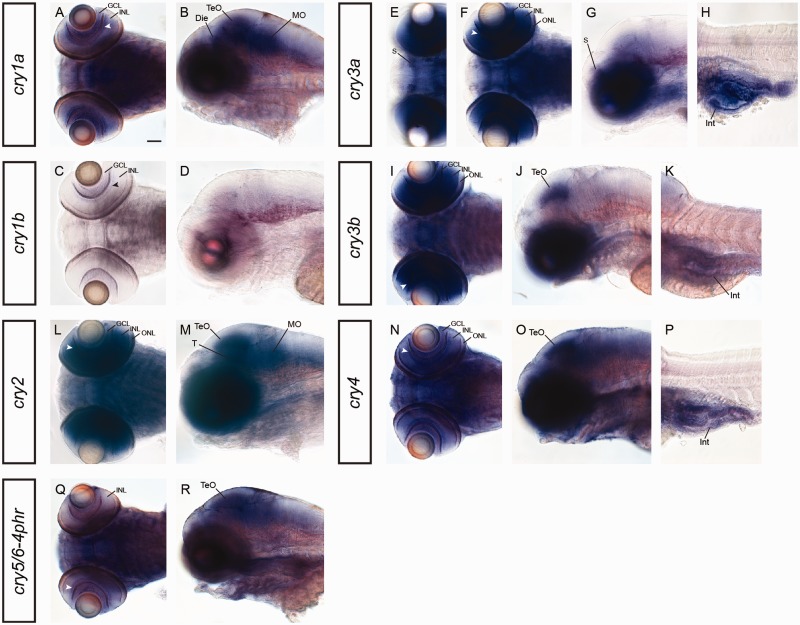
mRNA expression of *cryptochromes* in 5-day-old zebrafish. Expression of *cry1a* and *cry1b* in dorsal (*A*, *C*) and lateral (*B*, *D*) view. Although both riboprobes label the inner part of the retinal INL (arrowhead in *A* and *C*) and the GCL, *cry1a* shows a broad expression in the mid- (Die, TeO) and the hindbrain (MO). Dorsal (*E*, *F*, *I*) and lateral (*G*, *H*, *J*, *K*) view of *cry3a* and *cry3b* mRNA expression reveals paralog-specific labeling of *cry3a* in the subpallium (S in *E* and *G*) and of *cry3b* in the optic tectum (TeO in *J*). Both *cry3* paralogs label retinal layers (ONL, INL, GCL) including the inner part of the INL (arrowhead in *F* and *I*) and the intestine (Int in *H* and *K*). *cry2* expression in a dorsal (*L*) and lateral (*M*) view shows broad labeling in the retina (ONL, INL and inner part of INL [arrowhead], GCL), the midbrain (TeO, *T*), and the hindbrain (MO). *cry4* mRNA expression in dorsal (*N*) and lateral (*O*, *P*) view. Broad labeling is found all over the central nervous system but specifically in the inner part of the retinal INL (arrowhead in *N*) and in the optic tectum (TeO). In addition, *cry4* is expressed in the intestine (Int). Expression of *cry5/6-4phr* in dorsal (*Q*) and lateral (*R*) view indicates labeling in the inner part of the INL (arrowhead in *N*) and weakly in broad regions of the brain with a more intense staining in the optic tectum (TeO). Die, diencephalon; GCL, ganglion cell layer; INL, inner nuclear layer; Int, intestine; MO, medulla oblongata; ONL, outer nuclear layer; TeO, optic tectum; S, subpallium. Scale bar in (*A*) (corresponds to all images *A–R*) = 50 µm.

In order to reveal evolutionary events such as a division of function among ohnologs, we investigated the expression of *cry* transcript in 5-day-old zebrafish larvae. This is a developmental stage where the larvae possess a fully functional visual system and start to actively capture prey (Gestri et al. 2012). In the retina, both *cry1* paralogs are expressed in the inner part of the inner nuclear layer (INL) (arrowheads in fig. 4*A* and *C*), as well as in the ganglion cell layer (GCL) (fig. 4*A* and *C*). An additional expression in the diencephalon, the optic tectum, and the medulla oblongata (fig. 4*B*) was only detected with the *cry1a* riboprobe. The *cry3* paralogs, are both highly abundant in all retinal cell layers including the outer nuclear layer (ONL) where photoreceptors are located, and in the intestine (fig. 4*F*, *H*, *I*, and *K*), however, expression of *cry3a* in the subpallium and of *cry3b* in the optic tectum is paralog-specific (fig. 4*E*, *G*, and *J*).

*crys 2, 4,* and *5*, all belonging to the type I Cryptochromes, are expressed in various parts of the central nervous system (fig. 4*L*–*R*). The broadest expression is found for *cry2* transcripts which are expressed in all three nuclear cell layers ofthe retina (fig. 4*L*) as well as in the optic tectum, the tegmentum, and the medulla oblongata (fig. 4*M*). *cry4* and *cry5/6-4phr* show a very broad expression throughout the central nervous system (fig. 4*N*, *O*, *Q*, and *R*). However, for *cry4* we find a specific staining in all nuclear retinal layers and in the optic tectum (fig. 4*N*) and an additional staining in the intestine (fig. 4*P*). Retinal expression of *cry5/6-4phr* is confined to the inner part of the INL (arrowhead fig. 4*Q*). Although weak expression is found all over the brain, it appears slightly stronger in the optic tectum (fig. 4*R*). The developmental expression of the cry genes is summarized in table 3.

### Daily Expression Analysis of *crys* in the Retina of Adult Zebrafish

Because Crys might be involved in both visual and nonvisual processes that take place in the retina, we focused specifically on the eye and investigated the expression of *cry* genes in zebrafish eyes over a period of 24 h. As measuring mRNA levels in whole eyecups would not provide information about cyclic transcript expression in specific retinal cell layers, we combined this qRT-PCR analysis with ISH in adult retinal sections to generate more detailed information about oscillating *cryptochrome* expression.

**Fig. 5.—:**
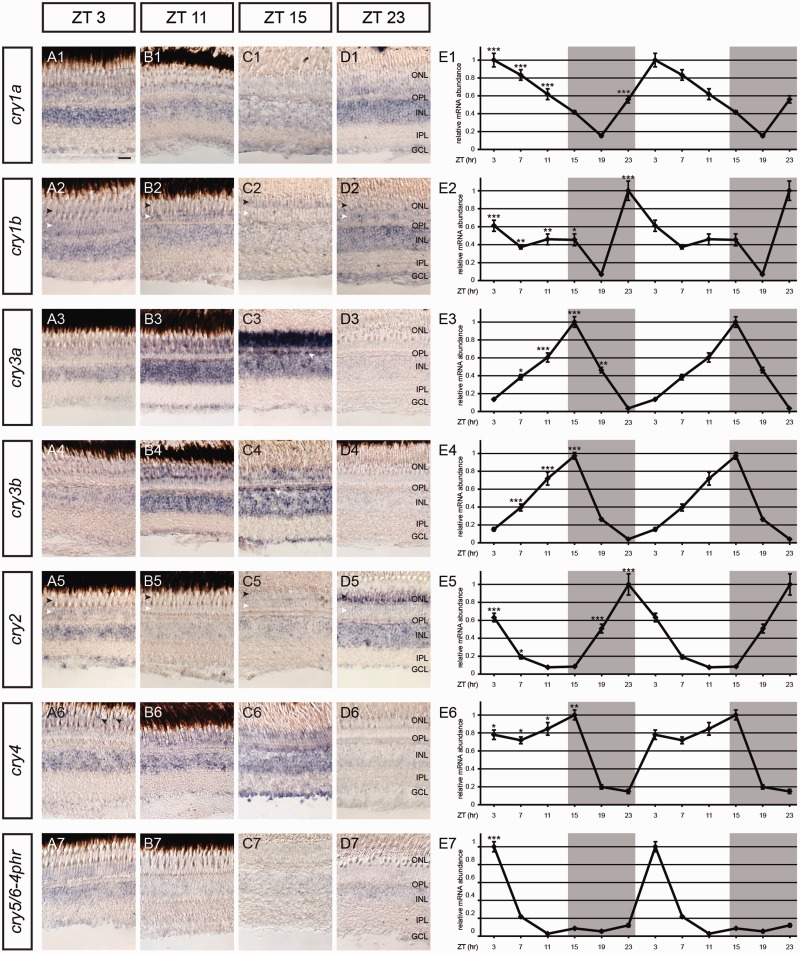
Daily mRNA expression pattern of *cryptochromes* in adult zebrafish retinas. (*A–D*) (1–7) ISH using different *cry* riboprobes on radial sections of the adult zebrafish retina at different ZTs indicated on top. Black arrowheads in (*A*2–*D*2) and (*A*5–*D*5) indicate the location of cone somata and white arrowheads the location of rod somata. White arrowheads in (*C*3) and (*C*4) point on the outermost part of the INL where horizontal cells are located. Black arrowheads in (*A*6) reveal specific staining in cone subtypes. (*E*1–*E*7) Double plot of qRT-PCR analysis showing daily expression of zebrafish *cry* transcripts in adult retinal tissue. Overall, qRT-PCR results confirm oscillating retinal transcript expression and show statistically significant daily variations in expression as shown by one-way ANOVA (*P* < 0.05) and Tukey’s post-hoc test. Asterisks mark significantly higher values relative to the lowest value (*P < 0.01, **P < 0.01, ***P < 0.001). The qRT-PCR values were averaged from three independent samples. Gray shading in qRT-PCR graphs represents night (lights off). On the *x* axis, the time in ZT is given. GCL, ganglion cell layer; INL, inner nuclear layer; IPL, inner plexiform layer; ONL, outer nuclear layer; OPL, outer plexiform layer. Scale bar in (*A*1) (applies to all images [*A–D*] [1–7]) = 20 µm.

*cry1* paralogs are most abundantly expressed during morning hours right after (*cry1a*, fig. 5*E*1) or right before (*cry1b*, fig. 5*E*2) light onset. The expression of *cry1a* peaks at Zeitgeber time (ZT) 3 and declines steadily during the following hours. We obtained a similar result with the ISH analysis: The high expression of *cry1a* in all retinal layers that peaks at ZT3, declines until ZT15 before it increases again (fig. 5*A*1–*D*1). Similar to its paralog *cry1a*, *cry1b* is expressed in all nuclear retinal layers (fig. 5*A*2–*D*2) and the strongest expression is seen at ZT23 which is in accordance with the qRT-PCR result (fig. 5*E*2). Expression in the ONL at ZT3, 11, and 23 is only seen in the proximal ONL where rod somata are located (white arrowheads, fig. 5*A*2–*D*2). At ZT15, however, the *cry1b* transcripts are localized in the layer where cone nuclei are found (black arrowheads, fig. 5*A*2–*D*2).

*cry3a* transcripts are expressed in all retinal nuclear layers of adult fish. The ONL shows the most obvious oscillation pattern with a peak at ZT15 and a trough some hours later at ZT23 (fig. 5*A*3–*D*3). In contrast to that, strongest expression in the INL and the GCL is found at ZT11 (fig. 5*B*3). Both *cry3* paralogs show a similar total retinal transcript expression with a steady increase during the day, a peak at ZT15 and a decrease during the dark phase (fig. 5*E*3 and *E*4). The riboprobe of *cry3b* stains all retinal layers in an oscillating manner (fig. 5*A*4–*D*4). Although the rhythmic expression in the GCL overlaps with the qRT-PCR result and peaks at ZT15 (fig. 4*C*4), the staining in the ONL and the INL remains high between ZT11 and ZT15 before it decreases until it is barely visible anymore at ZT23 (fig. 5*A*4–*D*4). Interestingly, both *cry3* paralogs show an intense staining at ZT15 in the outermost part of the INL where horizontal cells are located (white arrowheads, fig. 5*C*3 and *C*4).

We find *cry2* transcript oscillation in various retinal cell layers. Interestingly, *cry2* expression in the ONL seems to oscillate cell type dependent: Although we find a weak expression in the proximal ONL where rod somata are located at ZT3 and ZT23 (white arrowheads, fig. 5*A*5–*D*5), expression is very prominent in cones, most likely short wavelength cones, at ZT23 but invisible at all other time points (black arrowheads, fig. 5*A*5–*D*5). Expression in the INL is also rhythmic but stays more or less constant around light onset between ZT23 and ZT3 and the GCL shows the highest cry2 transcript expression at ZT3. Overall, these results overlap with the total retinal transcript abundance of *cry2* which peaks at ZT23 before light onset, declines steadily, and reaches its base level in the evening between ZT11 and 15 around light offset (fig. 5*E*5).

The zebrafish *cry4* qRT-PCR analysis reveals a trough at ZT23 followed by an increase in transcript abundance in the light phase and a peak at ZT15 (fig. 5*E*6). The ISH analysis confirms this result as we detect an increasing retinal *cry4* expression in the ONL, the INL, and the GCL between ZT3 and ZT15 but found no expression at ZT23 (fig. 5*A*6–*D*6). In addition, we located *cry4* transcripts in a distinct part of the ONL where cone somata are located but only at ZT3 (black arrowheads, fig. 5*A*6). At later time points, the expression in the ONL is rather broad and is not confined to a specific cell type.

*cry5/6-4phr* transcripts are weakly expressed in all nuclear retinal layers peaking at ZT3 (fig. 5*A*7–*D*7), which is confirmed by the 24-h-expression profile (fig. 5*E*7).

## Discussion

An extensive phylogenetic analysis is indispensable for the understanding of gene family evolution. Several studies describe the phylogeny of Cryptochromes; however, they only include few species and give a rather broad overview (Kobayashi et al. 2000; Lin and Todo 2005; Oliveri et al. 2014). Hence, we embarked on an extensive analysis including more than 100 eumetazoan species to elucidate evolutionary events that shape their extant appearance.

### Cryptochrome Phylogeny Reveals Diverse Specification Events of Three Ancestral Genes

Overall, the number of cryptochrome genes among species varies considerably. Eumetazoan Cryptochrome genes originated from three ancestral genes in the last common Bilateria ancestor, namely Cry1/2/3, Cry4/5, and Cry6. Cryptochrome 6 was likely lost at the basis of the Chordate evolution, which explains the appearance of Cry6 in Echinodermata and various Protostomia but its absence in Tunicates and Cephalochordates. Cry6 is therefore an invertebrate-specific Cryptochrome although it has been lost independently in several invertebrate lineages. A series of WGDs in vertebrates (indicated as R1, R2, and R3 in fig. 2) led to an increase in the number of vertebrate Crys. Relieve from selective pressure in duplicates resulted in the inactivation of several orthologs and ultimate gene loss. This most likely happened for Cry3 at the base of the tetrapod clade, as no tetrapod harbors a Cry3 ortholog, whereas their close relatives, the coelacanthimorpha, still possess a Cry3 ortholog. As no WGD event occurred at the base of any invertebrate lineage, the full set of Cryptochromes in invertebrates consists of the three Cryptochromes already present in the earliest ancestor of all Bilateria. The division between the vertebrate Cry1s, 2s, and 3s and all invertebrate Cry1/2/3s is obvious. Interestingly, invertebrate Cry4/5 orthologs are closer related to the vertebrate Cry5s. This suggests that the DNA-repair function, which is a key feature of Cry5, was the ancient function of this Cry and that the function of Cry4 changed subsequently after the genome duplication and has no functional representative in present invertebrates. Surprisingly, the lancelet (*Branchiostoma floridae*) Cry4/5 orthologs group within the vertebrate Cry4 branch. However, as the lancelet possesses two Cry4 paralogs which likely are the result of a tandem or partial chromosomal duplication event, it might be possible that one of these genes has kept the function of Cry5, whereas the other paralog adapted a Cry4-like function.

Interestingly, some invertebrates have lost all Cryptochromes. This group is composed of a variety of lineages ranging from ascidia (*C. intestinalis*) and hemichordata (*Saccoglossus kovalevskii*) over nematodes (*Caenorhabditis elegans*) to myriapoda (*Strigamia maritima*). Conspicuously, most of these either lead a parasitic lifestyle, thus their survival relies on a host, or they live in darkness where neither light-induced DNA damage has to be repaired nor light can be used to entrain the circadian clock. Although these organisms possess specialized light-sensing cells for phototactic behavior (e.g., *C. intestinalis*: Tsuda et al. 2003; *C. elegans*: Ward et al. 2008), the described photoreceptive pigments have never been associated with circadian function or navigation. Thus, parasitic species which fully depend on the physiological features of their hosts may have lost reliance on this biological function. Likewise, species that only need to orient themselves marginally may have no use for these machineries and subsequently lost the involved genes. This hypothesis may even explain the scarcity of Cry genes in some vertebrate genomes, such as the Cyclostomata (*Petromyzon marinus*) with only a Cry1 have a parasitic lifestyle (Bergstedt and Swink 1995) and serpents that only possess Cry1 and Cry2 rather relay on olfaction and infrared sensing than on a highly developed visual system (reviewed in Campbell et al. [2002]). In this context, however, it is interesting that highly developed species as humans manage with only two Cry genes and bees that are famously known to possess complex navigational skills and a circadian clock only possess one single Cryptochrome of type II. Apparently, all necessary features of circadian and navigational signaling can also be accomplished with only few or even only one Cryptochrome.

Among the closest living relatives of vertebrates we find different patterns: Although tunicates either lost all Cryptochromes or kept two family members that group outside the Cry1/2/3 and the Cry4/5 group, Cephalochordata have retained a Cry1/2/3 ortholog that groups closer to their vertebrate relatives and possess two Cry4/5s. These paralogs most likely originate from a local chromosomal duplication and are named Cry4.1 and Cry4.2 as they group with the vertebrate Cry4s. Although phylogenetic analysis reveals that the relationship between Cry4.1 and 4.2 of the lancelets and their vertebrate orthologs is rather distant, lancelets are the only invertebrates with a Cry4 variant. Interestingly, the divergences within the Cry4 clade are quite massive as the homology between Cry4.1 of *Branchiostoma* and Cry4 of chicken is only of 55% identity, whereas homologies in the Cry1/3 clade are usually bigger than 90%. This may suggest that the functional divergence of Cry4 is larger than the less sequence diverse type II Cry1.

The two WGD events at the basis of the vertebrate lineage increased the number of ancestral Cryptochromes to 8 of which 5 have been retained in Holostei and Teleostei such as the spotted gar (*Lepisosteus oculatus*) and in zebrafish (*Danio rerio*). Cry3 seems to have been lost in the tetrapod lineage as soon as they have diverged from the teleost lineage as no tetrapod species has maintained a Cry3 variant. All four remaining Crys were only kept by few tetrapods, namely amphibians, testudines, and iguania. An open question is why these lineages kept that many Cryptochromes compared with serpents or mammals that only harbor Cry1 and Cry2? One explanation may be found in the navigational use of Crys. Some amphibians have been shown to possess a light-dependent sensitivity toward a magnetic field that helps them to orient (Phillips and Borland 1994; Phillips et al. 2001; Diego-Rasilla et al. 2010) and also migrating turtles make use of the earth’s magnetic field, although most likely in a light-independent manner (reviewed in Wiltschko R and Wiltschko W [2012]). The proposed molecule involved in the radical pair mechanism—one of the hypothesized mechanisms how the earth’s magnetic field could be detected (Ritz et al. 2000; Rodgers and Hore 2009)—is Cryptochrome (reviewed in Liedvogel and Mouritsen [2010]). It is currently not known whether any Cryptochrome of amphibians or testudines is able to sense the magnetic field. Studies involving the type II Crys of humans (Foley et al. 2011) and the monarch butterfly (Gegear et al. 2010) have revealed magnetoreceptive abilities of these molecules. In addition, one of the key biophysical features of the radical-pair mechanism, the ability to form long-living radical pairs, has been shown in vitro for the bird’s Cry1 (Liedvogel et al. 2007; reviewed in Mouritsen and Hore [2012]). As these studies involve a broad range of organisms, the capability of Cryptochromes to detect magnetic fields is likely an ancient function and should therefore be preserved in different Cry subgroups. In addition, various convincing experiments that proof compass orientation using a magnetic field have already been conducted in migratory birds such as the European robin (Nießner et al. 2013) or the warbler (Mouritsen et al. 2004; Fusani et al. 2014). As birds do only possess the type II Crys 1 and 2 and the type I Cry4, Cry4 is the most prominent candidate for being involved in magnetoreception in higher vertebrates. The physiological function of Cry4 is currently not known, though it is established that the protein undergoes a blue/UV-light-dependent structural change in a retinal soluble fraction, the basis for serving as a photo- or magnetoreceptor (Watari et al. 2012). However, this function most likely operates through an unknown mechanism, as Cry4 does not interfere with CLOCK-BMAL1-mediated transcription known for all type II cryptochromes (Kobayashi et al. 2000; Kubo et al. 2006; Takeuchi et al. 2014).

Although it seems beneficial for every organism to keep a molecule with photolyase activity as Cry5*/6-4PhR* (Todo, Kim, et al. 1997; Todo, Ryo, et al. 1997; Uchida et al. 1997; Kobayashi et al. 2000), this gene was lost independently in several vertebrate lineages. The advantage of having a UV-induced DNA-repair mechanism in species that develop in a protective egg-shell or inside the mother’s uterus is probably low, which could give a rationale why mammals, snakes or birds have lost Cry5. However, turtle and alligator, both oviparous species, still contain *cry5*. In zebrafish, Cry5*/6-4PhR* function has been shown to have a strong impact on embryonic survival (Tamai et al. 2004) and a photorepair system seems to be present in fish (Dong et al. 2007), whereas a direct link between repair activity and viability is still lacking. The spike-like peak around midday that we report here for the zebrafish retinal *cry5/6-4phr* transcript expression may be adaptive for higher UV levels at noon.

Invertebrates never possess more than one ortholog of a Cry4/5 descendant. Interestingly, Cry5 is still present in all investigated mollusks and many insects. We do not know whether Cry5 still works as (6-4) photoproduct repair enzyme in these organisms, as shown in *Drosophila* (Todo et al. 1993; Todo, Ryo, et al. 1997; Selby and Sancar 2012).

Teleost fish harbor the largest number of Crys among all vertebrates due to the TGD event (indicated as R3 in fig. 2). This makes them an interesting lineage to study Cryptochrome evolution and phylogeny. Although Cry1 and Cry2 are both grouped as type II Cryptochromes and thus per definition inhibit CLOCK:BMAL1 mediated transcription, the zebrafish Cry2 is not able to do so (Kobayashi et al. 2000). Instead, the two zebrafish Cry3 paralogs that are closer related to Cry1 and were lost in the early lineage of tetrapods still show this type II Cry feature (Kobayashi et al. 2000). Among all species that underwent a third round of WGD, only two of all considered teleost fish, zebrafish and cavefish, sill retained both copies of Cry3, suggesting a specific function for the originally redundant copies in these species. Overall, both zebrafish and cavefish still bear exactly the same Cryptochrome members, which is not surprising as they are closely related. As all teleost fish only retained one copy of Cry2 and Cry5, the second paralogs were most likely lost already at the basis of the teleost lineage shortly after the duplication event, an outcome that is common for duplicated genes (Glasauer and Neuhauss 2014).

### Duplicated Paralogous Zebrafish *cryptochromes* Show Evolutionary Events

In order to gain insight into possible evolutionary processes of paralogous *cryptochrome* genes, we focused on the zebrafish. Using this model organism has two advantages: First, the ease of expression analysis of the full complement of the Cryptochrome family in a transparent vertebrate, and second the discovery of possible evolutionary events such as sub- and/or neofunctionalization as well as dosage balance (Force et al. 1999; Glasauer and Neuhauss 2014) by comparing expression of the two paralogous gene sets.

At first glance, a large difference in expression of the *cry1* paralogs is obvious: Although *cry1a* is expressed in broad areas of the CNS, *cry1b* is only marginally expressed in some retinal layers. The specific expression of *cry1a* in brain areas suggests subfunctionalization events, meaning that the function of the ancestral Cry1 in the brain was fully transferred to Cry1a. *cry1b* is still expressed, although at low levels, in the same retinal layers as its paralog. Expression of paralogous genes in the same areas may indicate dosage effects, implying that both paralogs need to be expressed to provide sufficient amounts of required protein. This is often found for genes expressing proteins involved in signaling pathways or proteins that form stoichiometric complexes (Conant and Wolfe 2007).

*cry3* paralogs are highly abundant in the eye and the intestine. Their identical transcript expression in the adult retina may suggest a similar function in the eye; however, mRNA expression analysis on adult retinal sections clearly shows differences in the oscillation pattern in the photoreceptor layer. In order to distinguish between sub- and neofunctionalization, a comparative expression analysis in different vertebrates would be necessary. Such data are currently missing, as most expression data obtained from other species are based on qRT-PCR analysis (e.g., Thompson et al. 2003) or ISH analyses of nonretinal tissue, such as the suprachiasmatic nucleus (e.g., Sumová et al. 2003). Hence, an assessment of functionalization events must await more comparative data.

### Circadian Aspects of Cryptochromes

We found that some invertebrates only harbor the above-mentioned type I Cry6, others have a type II Cry1 and that a third group even features one representative of both groups. The different biochemical features of these two subgroups are reflected in the way the endogenous clocks are built: Hymenopterans but also *Tribolium* that only possess a type II Cry1 most likely only harbor an internal core clock similar to mammals (Kume et al. 1999; Bertossa et al. 2014), whereas flies that only feature a type I form have cell-autonomous external clocks mediated through the photosensitive Cryptochrome 6 (Emery et al. 2000). However, the type I and invertebrate specific Cry6 has been shown to not only fulfill its role as circadian photoreceptor but in addition is part of the core clock (Ivanchenko et al. 2001; Krishnan et al. 2001; Levine et al. 2002), suggesting a tissue-specific involvement of this Cryptochrome in circadian signaling. Whether this holds also true for other Cry subtypes is currently unknown. A third group of invertebrates such as Lepidopterans (e.g., *Danaus plexipus*) feature both types of Crys and it is hypothesized that both engage their proposed function either as circadian photoreceptor or as repressor of the core-clock feedback loop (Zhu et al. 2008). It seems that this constitutes the ancestral state, as organisms from different arthropod classes still comprise both versions. Zebrafish possess not only type II but also type I Cryptochromes (Kobayashi et al. 2000). Although only the involvement of Cry1a in circadian processes has been shown (Tamai et al. 2007), possibly other Crys are involved in such processes as well. Most likely they compose the role of the external photoreceptor of each cells’ own circadian clock but also participate in internal clock mechanism similar to the mammalian Crys.

Many physiological functions of the retina such as visual sensitivity or the shedding of photoreceptor outer segments show a circadian pattern (reviewed in Guido et al. [2010]), strongly arguing for the existence of an autonomous oscillator in the retina (reviewed in Green and Besharse [2004] and Tosini et al. [2008]). Although there is evidence for the oscillator to be located in photoreceptors (Cahill and Besharse 1993; Pierce et al. 1993; Thomas et al. 1993; Tosini et al. 2007) or in the inner retina (Garbarino-Pico et al. 2004; Ruan et al. 2008), recent publications rather point toward a more complex picture and argue for an organization with several tissue-specific oscillators located in different retinal layers (e.g., Witkovsky et al. 2003; Dinet et al. 2007; Liu et al. 2012; Buhr and Van Gelder 2014; reviewed in Guido et al. [2010]). The observation of basically all *cry* gene transcripts in various retinal layers of different vertebrates (e.g., teleosts: Kobayashi et al. 2000; Velarde et al. 2009; frog: Zhu and Green 2001; birds: Kubo et al. 2006; mammals: Kamphuis et al. 2005; Miyamoto and Sancar 1998) makes them a valuable candidate for a contribution in one of these retinal oscillators. The varying oscillation patterns in different retinal cell layers we found for each *cry* strongly support the hypothesis of several independent clock mechanisms in the retina and reveal the importance of cell type specific analyses. More surprising is the very specific change in expression of *cry1b* and *cry2* throughout the day in the ONL, suggesting different functions in different subsets of photoreceptor cell types and again supporting the hypothesis of distinct retinal clocks.

Gene expression of both *cry1* paralogs in the retina seems to be at least partially regulated by a circadian mechanism as the changes in expression levels occur during the light or the dark phase but not upon changes in illumination. *cry1* transcripts of total retinae of rats and chicken stay cyclic in DD conditions (Haque et al. 2002; Sandu et al. 2011), which points toward circadian regulated gene expression. As chicken *cry1* and zebrafish *cry1a* expression can be induced by light (Haque et al. 2002; Tamai et al. 2007), at least nonmammalian vertebrate Cry1(a)s seems to be regulated by both a circadian oscillator and light. The zebrafish Cry1a has been found to be the light sensor of the cell-autonomous circadian clock in zebrafish (Tamai et al. 2007) and it possibly also bears this function in the eye. Nevertheless, another function within a circadian clock is also expected, as both *cry1* paralogs can inhibit CLOCK:BMAL1 induced transcription similar to the mammalian Crys (Kobayashi et al. 2000). The zebrafish Cry1a might has a dual function within the retina as circadian photoreceptor in one retinal layer and within a feedback loop in another. *cry1b* is only marginally expressed in the larval retina but shows high and oscillating expression in the adult retina in all nuclear layers, suggesting a developmentally regulated function.

In contrast to the *cry1* paralogs, both *cry3* paralogs as well as *cry2* and *cry4* show a light-dependent oscillation pattern with either a decrease or an increase in transcript expression around the light-dark transition. Although this suggests light-dependent regulation, in darkness living cavefish have degenerated eyes and a light-independent clock (Cavallari et al. 2011) whereas their genome still contains all these genes. Hence there may be another, light-independent function for these Cryptochromes outside of the retina. Cry4 for example has been shown to still possess the ability of blue-light absorption (Kubo et al. 2006; Ozturk et al. 2009) but whether it still executes this function in vivo is not known.

## Conclusion

This work provides the first broad overview of Cryptochrome phylogeny, a family of light sensitive proteins that are involved in the regulation of the circadian clock, light perception, and DNA-repair. The investigation of *cry* sequences from a variety of over 100 Bilateria led us to draw a comprehensive picture of Cryptochrome evolutionary history and to gain new insight into possible evolutionary events in the different invertebrate and vertebrate lineages. Based on our phylogeny, we hypothesize that in ancestral Bilateria three Cryptochromes Cry1/2/3, Cry4/5, and Cry6 built the basis for all currently known family members. WGD events and subsequent loss of some paralogous genes in the vertebrate lineage have shaped the Cryptochrome family in each vertebrate lineage. The base of the invertebrate lineages lacks such duplication events and the pattern of retained and lost *cry* genes can in some cases be correlated with ecology of these species. The presented comparative phylogeny may help to generate hypothesis about functional properties. The presented expression analysis of *cry* genes in zebrafish eyes forms the basis for a detailed analysis of Cryptochrome function in light-dependent DNA-repair, light sensing, and the circadian clock. The cyclic expression pattern of all *cry* transcripts in zebrafish retinal layers argues for an involvement in retinal circadian processes and the presence of several autonomous circadian clocks in the vertebrate retina.

## Supplementary Material

Supplementary table S1 is available at *Genome Biology and Evolution* online (http://www.gbe.oxfordjournals.org/).

Supplementary Data
