# Supplementary material for: Eumetazoan Cryptochrome Phylogeny and Evolution
Source: Genome Biol Evol. 2015 Jan 18;7(2):601–19. doi: 10.1093/gbe/evv010 (PMC4350181; doi:10.1093/gbe/evv010)
Supplement: Supplementary Data [file supp_7_2_601__index.html]

Eumetazoan Cryptochrome Phylogeny and Evolution — Supplementary Data 

# Eumetazoan Cryptochrome Phylogeny and Evolution

## Supplementary Data

files

**Files in this Data Supplement:**

- Supplementary Data - pdf file
- Supplementary Data - docx file
