# Supplementary material for: Eumetazoan Cryptochrome Phylogeny and Evolution
Source: Genome Biol Evol. 2015 Jan 18;7(2):601–19. doi: 10.1093/gbe/evv010 (PMC4350181; doi:10.1093/gbe/evv010)
Supplement: Supplementary Data [file supp_evv010_Haug_Supplementary_table.pdf]

Species (scientific name)	Abbreviation	Species (common name)	Name in study	Name(s) in databases	Accession # gene	Accession # transcript	Accession # protein	Identical to manual assembly	Coverage	Remarks
Adineta vaga	ava	Rotifer	no crys							
Aedes aegypti	aae	Yellow fever mosquito	Cry1	DNA photolyase	AaeL_AAEL011967	XM_001655728.1	XP_001655778.1	yes	Full	
			Cry5	DNA photolyase	AaeL_AAEL001175	XM_001658145.1	XP_001658195.1	yes	Full	
			Cry6	DNA photolyase	AaeL_AAEL004146	XM_001648448.1	XP_001648498.1	yes	Full	
Anolis carolinensis	aca	Green anole lizard	Cry1	cryptochrome 1 (photolyase-like) CRY1	LOC100553773 ENSACAG00000015184	XM_003220922.2 ENSACAT00000015310	XP_003220970.1 ENSACAP00000015006	yes	Full	
			Cry2	cryptochrome 2 (photolyase-like) CRY2	LOC100559432 ENSACAG00000014653	XM_003214641.2 ENSACAT00000014738	XP_003214689.1 ENSACAP00000014443	no	Full	
			Cry4	cryptochrome1-like / Uncharacterized protein	LOC100555537 ENSACAG00000004583	XM_008109915.1 ENSACAT00000004596	XP_008108122.1 ENSACAP00000004493	yes	Full	
			Cry5	cryptochrome1-like / Uncharacterized protein	LOC100565395 ENSACAG00000028672	XM_003225714.2 ENSACAT00000029262	XP_003225762.1 ENSACAP00000021844	yes	Full	
Acyrthosiphon pisum	api	Pea aphid	Cry1	--	ABLF02009467.1 ACYPI006584	-- ACYPI006584-RA	-- ACYPI006584-PA	yes	partial	
			Cry5	--	ABLF02035823.1 ACYPI003810	-- ACYPI003810-RA	-- ACYPI003810-PA	yes	Full	
			Cry6		ABLF02032292.1 ACYPI005768	-- ACYPI005768-RA	-- ACYPI005768-PA	no	Full	
Anopheles gambiae	aga	African malaria mosquito	Cry1	cryptochrome 2 (CRY2)	NT_078266.2 AGAP004261	DQ219483.1 AGAP004261-RA	ABB29887.1 AGAP004261-PA	yes	Full	
			Cry5	AgaP_AGAP008651	NT_078268.4 AGAP008651	XM_314748.3 AGAP008651-RA	XP_314748.3 AGAP008651-PA	yes	Full	
			Cry6	cryptochrome 1 (CRY1)	NT_078266.2 AGAP001958	DQ219482.1 AGAP001958-RA	ABB29886.1 AGAP001958-PA	yes	Full	
Astyanax mexicanus	ame	Mexican cave fish	Cry1a	cryptochrome 1a (CRY1a)	APWO01031161.1 ENSAMXG00000005406	KF737847.1 ENSAMXT00000005553	AHA91701.1 ENSAMXP00000005553	no	partial	
			Cry1b	cryptochrome 1b (CRY1b); cryptochrome-1-like	103041767 ENSAMXG00000020084	XM_007250531.1 ENSAMXT00000020700	XP_007250593.1 ENSAMXP00000020700	no	partial	
			Cry2	cryptochrome 3; cryptochrome-2-like	103044559 ENSAMXG00000009568	XM_007237111.1 ENSAMXT00000009833	XP_007237173.1 ENSAMXP00000009833	no	partial	
			Cry3a	cryptochrome 2a (CRY2a)	APWO01106043-45 ENSAMXG00000000150	-- ENSAMXT00000000154	-- ENSAMXP00000000154	no	partial	
			Cry3b	cryptochrome 2b (CRY2b)	103034515 ENSAMXG00000008895	XM_007256752.1 ENSAMXT00000009162	XP_007256814.1 ENSAMXP00000009162	no	partial	
			Cry4							
			Cry5	cryptochrome 5 (CRY5); cryptochrome-1-like	103034890 ENSAMXG00000006386	XM_007244716.1 ENSAMXT00000006552	XP_007244778.1 ENSAMXP00000006552	yes	Full	
Alligator mississippiensis	ami	American alligator	Cry1	cryptochrome-1-like	AKHW01071470-74	XM_006271660.1	XP_006271722.1	yes	Full	
			Cry2	cryptochrome 2 (photolyase-like) CRY2	AKHW01085219-20	XM_006274071.1	XP_006274133.1	yes	Full	
			Cry5	cryptochrome-1-like	AKHW01093963.1	XM_006275603.1	XP_006275665.1	yes	Full	
Aplysia californica	apca	Californian sea slug	Cry1	--	--	GBDA01071989.1	--	yes	Full	
			Cry5	--	NW_004797295.1	GBBE01037839.1 GBBE01077832.1	--	no	partial	
			Cry6	--	NW_004797278.1	GBCZ01053527.1	--	no	partial	
Apis mellifera	apme	Honey bee	Cry1	cryptochrome 2 (CRY2)	AADG06001305.1 GB10211	GAZV01036545.1 GB10211-RA	-- GB10211-PA	yes	Full	
Alligator sinensis	asi	Chinese alligator	Cry1	cryptochrome-1-like	102388600	XM_006024277.1	XP_006024339.1	yes	Full	
			Cry2	cryptochrome 2 (photolyase-like) CRY2	102378706	XM_006026947.1	XP_006027009.1	yes	partial	
			Cry5	cryptochrome-1-like	102382870	XM_006021354.1	XP_006021416.1	yes	Full	
Atta cephalotes	atce	Leaf-cutting ant	Cry1	--	ADTU01021771.1 ACEP27227	-- ACEP27227-RA	-- ACEP27227-PA	no	Full	
Brachionus calyciflorus	bca	Rotifer	Cry5	--	--	GACQ01030869.1	--	yes	Full	
			Cry6	--	--	GACQ01022953.1	--	yes	partial	
Branchiostoma floridae	bfl	Lancet	Cry1	--	7248168	XM_002609455.1	XP_002609501.1	no	Full	
			Cry4a	--	7206346	XM_002595028.1	XP_002595074.1	yes	partial	
			Cry4b	--	7250214	XM_002609457.1	XP_002609503.1	no	partial	
Biomphalaria glabrata	bgl	Freshwater snail	Cry1	--	APKA01015257.1 APKA01042121.1	--	--	n.a	Full	
			Cry5	--	APKA01039721.1		--	n.a	partial	
			Cry6	--	APKA01092690.1	--	--	n.a	partial	
Bombyx mori	bmo	China silkworm	Cry1	cryptochrome 2 (CRY2)	100500922 BGIBMGA007789	NM_001195698.1 BGIBMGA007789-RA	NP_001182627.1 BGIBMGA007789-TA	yes/no	Full	
			Cry5	cryptochrome-1-like	101735529 BGIBMGA000221	XM_004931937.1 BGIBMGA000221-RA	XP_004931994.1 BGIBMGA000221-TA	no	Full	
			Cry6	cryptochrome 1 (CRY1)	100500923 BGIBMGA007140	NM_001195699.1 BGIBMGA007140-RA	NP_001182628.1 BGIBMGA007140-TA	yes/no	Full	
Bithynia siamensis	bsi	Freshwater snail	Cry1	--	--	GAGS01026551.1	--	yes	partial	
			Cry5	--	--	GAGS01026313.1	--	yes	Full	
			Cry6	--	--	GAGS01010240.1 GAGS01065309.1	--	yes	partial	
Bombus terrestris	bte	Bumblebee	Cry1	cryptochrome-1-like	100647270	XM_003398483.1	XP_003398531.1	yes	Full	
Botryllus schlosseri	bsc	Golden star tunicate	no crys							
Centruroides exilicauda	cex	Bark scorpion	Cry1	--	AXZI01030976-77	--	--	n.a	partial	
Canis familiaris	cfa	Dog	Cry1	cryptochrome 1 (photolyase-like) CRY1	474528 ENSCAFG00000001797	XM_005625937.1 ENSCAFT00000002826	XP_005625994.1 ENSCAFP00000002620	yes	Full	
			Cry2	cryptochrome 2 (photolyase-like) CRY2	483641 ENSCAFG00000009379	XM_540761.4 ENSCAFT00000014927	XP_540761.2 ENSCAFP00000013814	yes	Full	
Calanus finmarchicus	cfi	Zooplankton	Cry1	--	--	GAXK01199676.1	--	n.a	Full	
			Cry5	--	--	GAXK01107177.1	--	n.a	Full	
			Cry6a	--	--	GBFB01166903.1	--	n.a	Full	
			Cry6b	--	--	GBFB01092902.1	--	n.a	partial	
Ciona intestinalis	cin	Vase tunicate	no crys							
Crassostrea gigas	cgi	Pacific oyster	Cry1	Cleavage Factor 77	AFTI01019304.1 CGI_10006975	-- EKC20054	-- EKC20054	no	Full	
			Cry5	cryptochrome 1	AFTI01022078/79 CGI_10007760	-- EKC39908	-- EKC39908	no	partial	
			Cry6	cryptochrome 1	AFTI01030112.1 CGI_10017817	-- EKC26912	-- EKC26912	no	Full	
Callorhinchus milii	cmi	Ghost (Elephant) shark	Cry1	cryptochrome 1	103179904	XM_007895430.1	XP_007893621.1	yes	Full	
			Cry2	cryptochrome 2	103175031	XM_007887759.1	XP_007885950.1	yes	partial	
Chelonia mydas	cmy	Green sea turtle	Cry1	cryptochrome-1-like	102945802	XM_007066141.1	XP_007066203.1	yes	Full	
			Cry2	cryptochrome 2 (CRY2)	102947408	XM_007070839.1	XP_007070901.1	no	Full	
			Cry4	cryptochrome 1 (CRY1)	102945380	XM_007054312.1	XP_007054374.1	yes	Full	
			Cry5	cryptochrome-1-like	102947919	XM_007066377.1	XP_007066439.1	yes	Full	
Chrysemys picta bellii	cpb	Painted turtle	Cry1	cryptochrome 1 (CRY1)	101946418 ENSPSIG00000014000_1	XM_005300804.2 ENSPSIG00000014000_1	XP_005300861.1 ENSPSIG00000014000_1	yes/no	Full	
			Cry2	cryptochrome 2 (CRY2)	101952077 ENSPSIG00000012972_1	XM_005302233.1 ENSPSIG00000012972_1	XP_005302290.1 ENSPSIG00000012972_1	yes/no	Full	
			Cry4	cryptochrome-1-like	101945424 n.d	XM_008174731.1 n.d	XP_008172953.1 n.d	no	partial	
			Cry5	cryptochrome-1-like	101942922 ENSPSIG00000013409_1	XM_008171074.1 ENSPSIG00000013409_1	XP_008169296.1 ENSPSIG00000013409_1	yes/no	Full	
Chlamydomonas reinhardtii	cre		CryA1	cryptochrome photoreceptor	5723611	XM_001698002.1	XP_001698054.1	yes	Full	
Capitella teleta	cte	Polychaete worm	Cry1	--	AMQN01001386.1 CapteG178510	-- CapteT178510	-- CapteP178510	no	Full	
			Cry6	--	AMQN01005339.1 CapteG226189	-- CapteT226189	-- CapteP226189	no	Full	
Dendroctonus ponderosae	dpo	Mountain pine beetle	Cry1	--	APGK01037204.1 YQE_06087	-- ENN77258	-- ENN77258	no	Full	
Dasypus novemcinctus	dno	Nine-banded armadillo	Cry1	cryptochrome 1 (photolyase-like) CRY1	101438819 ENSDNOG00000011730	XM_004473303.1 ENSDNOT00000011732	XP_004473360.1 ENSDNOP00000009091	yes/no	Full	
			Cry2	cryptochrome 2 (photolyase-like) CRY2	101444859 n.d	XM_004457886.1 n.d	XP_004457943.1 n.d	no	partial	
Drosophila melanogaster	dme	Fruit fly	Cry5	(6-4)-photolyase (phr6-4)	35322 FBgn0016054	NM_165334.3 FBtr0081404	NP_724274.1 FBpp0080934	yes	Full	
			Cry6	cryptochrome (cry)	42305 FBgn0025680	NM_169852.1 FBtr0083736	NP_732407.1 FBpp0083150	yes	Full	
Danaus plexippus	dpl	Monarch butterfly	Cry1	cryptochrome 2 (CRY2)	AGBW01003794.1 KGM_05008	DQ184682.1 EHJ74426	ABA62409.1 EHJ74426	yes	Full	
			Cry5	(6-4)-photolyase (phr6-4)	AGBW01003313.1 KGM_20382	EF117813.1 EHJ75225	ABO38436.1 EHJ75225	yes/no	Full	
			Cry6	cryptochrome (cry)	AGBW01012954.1 KGM_06594	AY860425.1 EHJ63675	AAX58599.1 EHJ63675	yes	Full	
Daphnia pulex	dpu	Water flea	Cry1	CRY-M	ACJG01001137.1 DAPPUDRAFT_195559	-- EFX82092	-- EFX82092	no	Full	
			Cry5	DNA photolyase 1	ACJG01000698.1 DAPPUDRAFT_300463	-- EFX85418	-- EFX85418	no	partial	
			Cry6	CRY-D	ACJG01002273.1 DAPPUDRAFT_305792	-- EFX77441	-- EFX77441	no	partial	
Danio rerio	dre	Zebrafish	Cry1a	cryptochrome cicadian clock 1a (CRY1a)	100003956 ENSDARG00000045768	NM_001077297.2 ENSDART00000130692	NP_001070765.2 ENSDARP00000112452	yes	Full	
			Cry1b	cryptochrome cicadian clock 1b (CRY1b)	554836 ENSDARG00000011583	NM_131790.4 ENSDART00000128609	NP_571865.4 ENSDARP00000104471	yes	Full	
			Cry2	cryptochrome 3 (cry3); cryptochrome-2	83774 ENSDARG00000024049	NM_131786.2 ENSDART00000028606	NP_571861.2 n.d	yes/no	Full	
			Cry3a	cryptochrome 2a (CRY2a); cryptochrome-1	573209 ENSDARG00000069074	XM_005166892.1 ENSDART00000129210	XP_005166949.1 ENSDARP00000109829	yes	Full	
			Cry3b	cryptochrome 2b (CRY2b)	83780 ENSDARG00000091131	XM_005168278.1 ENSDART00000125347	XP_005168335.1 ENSDARP00000108036	yes	Full	
			Cry4	cryptochrome 4 (cry4)	83775 ENSDARG00000011890	XM_005168277.1 ENSDART00000105873	XP_005168334.1 ENSDARP00000096651	yes	Full	
			Cry5	cryptochrome 5 (cry5); z6-4phr	83776 ENSDARG00000019498	XM_005155405.1 ENSDART00000023831	XP_005155462.1 ENSDARP00000015621	yes	Full	

Ephemera danica	eda	Mayfly	Cry1	--	AYNC01059722-23		--		--		n.a	Full	
			Cry5	--	AYNC01074898.1		--		--		n.a	partial	
			Cry6	--	AYNC01051445-46		--		--		n.a	partial	
Eurytemora affinis	eaf	Zooplankton	Cry1	--	AZAI01050145-48		--		--		n.a	Full	
			Cry5	--	AZAI01023513-17		--		--		n.a	parial	
			Cry6a	--	AZAI01031425.1		--		--		n.a	Full	
			Cry6b	--	AZAI01003580-81		--		--		n.a	Full	
Echinococcus granulosus	egr	Dog tapeworm	no crys										
Frankliniella occidentalis	foc	Western flower thrips	Cry1	--	JMDY01025861-62		GAXD01023628.1		--		no	partial	
			Cry5	--	JMDY01020311.1		--		--		n.a	Full	
			Cry6	--	JMDY01029567.1		--		--		n.a	partial	
Gasterosteus aculeatus	gac	Three spined stickleback	Cry1a	cryptochrome 1 (photolyase-like) CRY1	AANH01009354.1	ENSGACG00000020037	--	ENSGACT00000026521	--	ENSGACP00000026470	no	Full	
			Cry1b	cryptochrome 1b (CRY1b)	AANH01001691.1	ENSGACG00000013480	--	ENSGACT00000017852	--	ENSGACP00000017817	no	partial	
			Cry2	cryptochrome 3 (cry3)	AANH01008624.1	ENSGACG00000002398	--	ENSGACT00000003153	--	ENSGACP00000003142	no	Full	
			Cry3	cryptochrome 2a (CRY2a)	AANH01002621.1	ENSGACG00000010460	--	ENSGACT00000013860	--	ENSGACP00000013835	no	Full	
			Cry5	cryptochrome 5 (cry5)	AANH01000285.1	ENSGACG00000016441	--	ENSGACT00000021748	--	ENSGACP00000021707	no	Full	
Gallus gallus	gga	Chicken	Cry1	cryptochrome 1 (photolyase-like) CRY1	374093	ENSGALG00000012638	NM_204245.1	ENSGALT00000020627	NP_989576.1	ENSGALP00000020598	yes/no	Full	
			Cry2	cryptochrome 2 (photolyase-like) CRY2	374092	ENSGALG00000008436	NM_204244.1	ENSGALT00000013745	NP_989575.1	ENSGALP00000013730	yes	Full	
			Cry4	cryptochrome 4 (cry4)	395100	ENSGALG00000000104	NM_001039596.1	ENSGALT00000000143	NP_001034685.1	ENSGALP00000000142	yes	Full	
Glycine max	gma	Soybean	CryP1	(6-4)DNA photolyase-like	100780347		XM_003531652.2		XP_003531700.2		yes	Full	
Halyomorpha halys	hha	Brown marmorated stink bug	Cry1	--	JMPT01055744-45		--		--		n.a	partial	
Homalodisca vitripennis	hvi	Glassy-winged sharpshooter	Cry1	--	JJNS01059765-73		--		--		n.a	partial	
			Cry5	--	JJNS01230488-97	JJNS01368762.1	--		--		n.a	partial	
			Cry6	--	JJNS01076682.1		--		--		n.a	Full	
Helobdella robusta	hro	Leech	no crys										
Homo sapiens	hsa	Human	Cry1	cryptochrome cicadian clock 1 (CRY1)	1407	ENSG00000008405	NM_004075.4	ENST00000008527	NP_004066.1	ENSP00000008527	yes	Full	
			Cry2	cryptochrome cicadian clock 2 (CRY2)	1408	ENSG00000121671	NM_021117.3	ENST00000443527	NP_066940.2	ENSP00000406751	yes	Full	
Hordeum vulgare	hvu	Cereal grain	CryP1	--	CAJW010066338.1	CAJX010878537.1	AK360897.1		BAJ92104.1		yes	Full	
Hymenolepis microstoma	hmi	Rodent tapeworm	Cry5										
Ixodes scapularis	isc	Black-legged tick	Cry1	DNA photolyase	8036482	ISCW011472	XM_002411857.1	ISCW011472-RA	XP_002411902.1	ISCW011472-PA	no	partial	
Ladona fulva	lfu	Scarce Chaser	Cry1	--	APVN01145033-34		--		--		n.a	partial	
			Cry5	--	APVN01051671-72		--		--		n.a	Full	
			Cry6	--	APVN01084692.1		--		--		n.a	parial	
Loxodonta Africana	laf	African savanna elephant	Cry1	cryptochrome 1 (photolyase-like) CRY1	100674579	ENSLAFG00000014082	XM_003405313.1	ENSLAFT00000014082	XP_003405361.1	ENSLAFP00000011792	no	Full	
			Cry2	cryptochrome 2 (photolyase-like) CRY2	100668359	ENSLAFG00000027340	XM_003412109.1	ENSLAFT00000027247	XP_003412157.1	ENSLAFP00000020487	yes/no	Full	
Lethenteron camtschaticum	lca	Arctic lamprey	Cry1										
Latimeria chalumnae	lch	Coelacanth	Cry1	cryptochrome 1 (photolyase-like) CRY1	102346958	ENSLACG00000008174	XM_005989252.1	ENSLACT00000009338	XP_005989314.1	ENSLACP00000009267	no/yes	Full	
			Cry2	cryptochrome 2 (photolyase-like) CRY2	102356350	ENSLACG00000018488	XM_005986968.1	ENSLACT00000021183	XP_005987030.1	ENSLACP00000021043	no	partial	
			Cry3	cryptochrome-1-like	102365364	ENSLACG00000010538	XM_005987685.1	ENSLACT00000012065	XP_005987747.1	ENSLACP00000011974	yes/no	Full	
			Cry4	cryptochrome 4 (cry4)	--	ENSLACG00000012369	GAAA01026677.1	ENSLACT00000014152	--	ENSLACP00000014053	no	Full	
Leucoraja erinacea	ler	Little skate	Cry1	--	AESE010673288.1		--		--		n.a	partial	
			Cry2	--	AESE011547252.1		--		--		n.a	partial	
Lottia gigantea	lgi	Owl limpet	Cry1	--	AMQO01005011.1	LotgiG131547	--	LotgiT131547	--	LotgiP131547	no	Full	
			Cry5	--	AMQO01006184-85	LotgiG180057	--	LotgiT180057	--	LotgiP180057	no	Full	
			Cry6	--	AMQO01007909.1	LotgiG143281	--	LotgiT143281	--	LotgiP143281	no	Full	
Lepisosteus oculatus	loc	Spotted Gar	Cry1	cryptochrome-1-like; cryptochrome 1b (CRY1b)	102698691	ENSLOCG00000015272	XM_006633086.	ENSLOCT00000018825	XP_006633149.1	ENSLOCP00000018793	yes/no	Full	
			Cry2	cryptochrome-2-like; cryptochrome 3 (CRY3)	102691916	ENSLOCG00000014655	XM_006643120.1	ENSLOCT00000018079	XP_006643183.1	ENSLOCP00000018047	yes/no	Full	
			Cry3	cryptochrome-1-like; cryptochrome 2a (CRY2a)	102698939	ENSLOCG00000011417	XM_006628513.1	ENSLOCT00000014069	XP_006628576.1	ENSLOCP00000014040	no	Full	
			Cry4	cryptochrome-2-like; cryptochrome 4 (CRY4)	102682629	ENSLOCG00000011465	XM_006628517.1	ENSLOCT00000014118	XP_006628580.1	ENSLOCP00000014089	yes	Full	
			Cry5	cryptochrome-2-like; cryptochrome 5 (CRY5)	102688545	ENSLOCG00000002684	XM_006642227.1	ENSLOCT00000003178	XP_006642290.1	ENSLOCP00000003171	no	partial	
Limnephilus lunatus	llu	Caddisfly	Cry1	--	JDSM01035215-16		--		--		n.a	partial	
			Cry5	--	JDSM01436565.1		--		--		n.a	Full	
			Cry6	--									
Limulus Polyphemus	lpo	Horseshoe crab	Cry1	--	AZTN01015135-38		--		--		n.a	partial	
			Cry5	--	AZTN01088104-05		--		--		n.a	partial	
			Cry6	--	AZTN01152208-10		--		--		n.a	partial	pseudogene
Lepeophtheirus salmonis	lsa	Salmon louse	Cry6	--	ADND02024057.1		--		--		n.a	Full	
Lytechinus variegatus	lva	Green sea urchin	Cry1	--	GAUR01029447.1		--		--		n.a	partial	
			Cry5	--	AGCV01417937-41		GAUR01061578.1		--		n.a	Full	
			Cry6	--	AGCV01402035-38		GAUR01018405.1		--		n.a	Full	
Monodelphis domestica	md	Gray short-tailed opossum	Cry1	cryptochrome 1 (photolyase-like) CRY1	100619810	ENSMODG00000001579	XM_007503484.1	ENSMODT00000001968	XP_007503546.1	ENSMODP00000001926	no/yes	Full	
			Cry2	cryptochrome 2 (photolyase-like) CRY2	100618618	ENSMODG00000019881	XM_007497374.1	ENSMODT00000025247	XP_007497436.1	ENSMODP00000024807	yes	Full	
Musca domestica	mdo	House fly	Cry5	cryptochrome-1-like	101893478		XM_005182716.1		XP_005182773.1		yes	Full	
			Cry6	cryptochrome-1-like	101889464		XM_005178150.1		XP_005178207.1		yes	Full	
Mengenilla moldrzyki	mmo		Cry1	--	AGDA01005037.1	AGDA01038861.1	--		--		n.a	partial	
Mesobuthus martensii	mma	Chinese scorpion	Cry1	--	AYEL01085448-49		--		--		n.a	Full	
Mus musculus	mmu	House mouse	Cry1	cryptochrome 1 (photolyase-like) CRY1	12952	ENSMUSG00000020038	NM_007771.3	ENSMUST00000020227	NP_031797.1	ENSMUSP00000020227	yes	Full	
			Cry2	cryptochrome 2 (photolyase-like) CRY2	12953	ENSMUSG00000068742	NM_009963.4	ENSMUST00000090559	NP_034093.1	ENSMUSP00000088047	yes	Full	
Micromonas pusilla	mpu	Small eukaryotic alga	CryA1	--	9686404		XM_003061182.1		XP_003061228.1		yes	Full	
Metaseiulus occidentalis	moc	Western orchard predatory mite	no crys										
Ornithorhynchus anatinus	oan	Platypus	Cry1	cryptochrome 1 (photolyase-like) CRY1	100077381	ENSOANG00000005530	XM_007670116.1	ENSOANT00000008784	XP_007668306.1	ENSOANP00000008782	no	Full	
			Cry2	cryptochrome 2 (photolyase-like) CRY2	100075977	ENSOANG00000005350	XM_007668038.1	ENSOANT00000008519	XP_007666228.1	ENSOANP00000008517	no	partial	
Ophiophagus hannah	oha	King cobra	Cry1	cryptochrome 1 (CRY1)	AZIM01001837.1		--		--		n.a	Full	
			Cry2	cryptochrome 2 (CRY2)	AZIM01000886.1	AZIM01001807.1	--		--		n.a	Full	
Oryzias latipes	ola	Medaka	Cry1a	cryptochrome 1 (photolyase-like) CRY1	101167667	ENSORLG00000016410	XM_004083204.1	ENSORLT00000020543	XP_004083252.1	ENSORLP00000020542	yes/no	Full	
			Cry1b	cryptochrome 1b (photolyase-like) CRY1b	101160902	ENSORLG00000016382	XM_004069867.1	ENSORLT00000020515	XP_004069915.1	ENSORLP00000020514	yes/no	Full	
			Cry2	cryptochrome-2-like; CRY3	101173389	ENSORLG00000014701	XM_004070156.1	ENSORLT00000018427	XP_004070204.1	ENSORLP00000018426	no	Full	
			Cry3	cryptochrome-1-like; cryptochrome 2a (CRY2a)	101164536	ENSORLG00000019097	XM_004086308.1	ENSORLT00000023814	XP_004086356.1	ENSORLP00000023813	yes/no	Full	
			Cry5	cryptochrome-1-like; cryptochrome 5 (CRY5)	101174986	ENSORLG00000012462	XM_004067403.1	ENSORLT00000015605	XP_004067451.1	ENSORLP00000015604	yes/no	Full	
Oryza sativa	osa	Rice	CryP1	Similar to 6-4 photolyase; (6-4)DNA photolyase	4328669	OS02G0204400	NM_001052778.2	OS02T0204400-00	NP_001046243.2	OS02T0204400-00	yes	Full	
Ostreococcus tauri	ota	Green alga	CryA1	cryptochrome-1-like protein	9833287		XM_003079075.1		XP_003079123.1		yes	Full	
Pachypsylla venusta	pve	Hackberry blistergall psyllid	Cry1	--	AZLD01080911-18		GAOP01061315.1		--		n.a	Full	
			Cry5	--	AZLD01070407-15		GAOP01114724.1		--		n.a	Full	
			Cry6	--	AZLD01065515-23		GAOP01084159.1		--		n.a	Full	
Patiria miniata	pmi	Bat star	Cry1	--	AKZP01129361.1		GAWB01027325-36		--		no	partial	
			Cry5	--	AKZP01028794.1		GAWB01053884-85		--		no	partial	
			Cry6	--	AKZP01076536-37		GAWB01026752-53		--		no	Full	
Priapulus caudatus	pca	Priapulid worm	no crys										
Pediculus humanus corporis	phu	Body louse	Cry1	Deoxyribodipyrimidine photo-lyase	8235728	PHUM490060	XM_002430500.1	PHUM490060-RA	XP_002430545.1	PHUM490060-PA	no	Full	
Platynereis dumerilii	pdu	Dumeril's clam worm	Cry1										
			Cry5										
			Cry6										

Pontastacus leptodactylus	ple	Galician Crayfish	Cry1	--	--		GAFS01016708.1		--		yes	partial	
Petromyzon marinus	pma	Sea lamprey	Cry1	cryptochrome DASH	ENSPMAG00000004074		ENSPMAT00000004504		ENSPMAP00000004487		no	partial	
Python molurus	pmo	Indian python	Cry1	--	AEQU02105487-94		--		--		n.a	Full	
			Cry2	--	AEQU02146289.1		--		--		n.a	Full	
Pelodiscus sinensis	psi	Chinese softshell turtle	Cry1	cryptochrome-1-like; cryptochrome 1 (CRY1)	102443469	ENSPSIG00000014000	XM_006138065.1	ENSPSIT00000015866	XP_006138127.1	ENSPSIP00000015791	no	Full	
			Cry2	cryptochrome 2 (photolyase-like) CRY2	102450877	ENSPSIG00000012972	XM_006124501.1	ENSPSIT00000014684	XP_006124563.1	ENSPSIP00000014616	no	partial	
			Cry4	cryprochrome-1-like	102460658	ENSPSIG00000012858	XM_006115696.1	ENSPSIT00000014515	XP_006115758.1	ENSPSIP00000014447	yes/no	Full	
			Cry5	--	AGCU01007701-02	ENSPSIG00000013409	--	ENSPSIT00000015155	--	ENSPSIP00000015084	no	Full	
Parasteatoda tepidariorum	pte	Common house spider	Cry1	--	AOMJ01140962.1		--		--		n.a	partial	
			Cry5	--	AOMJ01119002.1		--		--		n.a	partial	
			Cry6	--	AOMJ01100131.1......		--		--		n.a	partial	
Ramulus artemis	rar	Vietnamese Stick Bug	Cry1										
			Cry5										
			Cry6										
Rhodnius prolixus	rpr	Kissing bug	Cry1	--	ACPB02032730-31		--		--		n.a	partial	
Saccoglossus kowalevskii	sko	Acorn worm	no crys										
Schistosoma mansoni	sma	Blood worm	no crys										
Stegodyphus mimosarum	smi	Social spider	Cry1	--	AZAQ01011524.1		--		--		n.a	partial	
			Cry5	--	AZAQ01115667-72		--		--		n.a	partial	
			Cry6	--	AZAQ01021104-06		--		--		n.a	partial	
Strigamia maritima	stma	European centipede	no crys										
Stylophora pistillata	spi	Hood coral	Cry1	--	--		GARY01007221.1		--		n.a	partial	
			Cry2	--	--		GARY01026968.1		--		n.a	partial	
			Cry5	--	--		GARY01010209.1		--		n.a	Full	
Strongylocentrotus purpuratus	spu	Sea Urchin	Cry1	cryptochrome-1-like	580742	SPU_007204	XM_780780.3	SPU_007204-tr	XP_785873.3	SPU_007204-tr	yes	parial	
			Cry5	cryptochrome-2-like	583959	SPU_019722	XM_783845.3	SPU_019722-tr	XP_788938.2	SPU_019722-tr	yes	Full	
			Cry6	cryptochrome-1-like	581225	SPU_000282	XM_781238.3	SPU_000282-tr	XP_786331.2	SPU_000282-tr	no	Full	
Solanum tuberosum	stu	Potato	CryP1	6-4 photolyase like	102605735	PGSC0003DMG40002862	XM_006346527.1	PGSC0003DMT400073683	XP_006346589.1	PGSC0003DMT400073683	yes/no	Full	
Tribolium castaneum	tca	Red flour beetle	Cry1	cryptochrome 2	661402	TCOGS2:TC010454	NM_001083325.1	TCOGS2:TC010454-RA	NP_001076794.1	TCOGS2:TC010454-PA	yes	Full	
Taeniopygia guttata	tgu	Zebra finch	Cry1	cryptochrome 1 (photolyase-like) CRY1	100223409	ENSTGUG00000010999	XM_002196518.2	ENSTGUT00000011476	XP_002196554.2	ENSTGUP00000011352	no	Full	
			Cry2	cryptochrome 2 (photolyase-like) CRY2	100224803	ENSTGUG00000010722	XM_002198864.2	ENSTGUT00000011186	XP_002198900.1	ENSTGUP00000011066	no	Full	
			Cry4	cryptochrome-1-like	100231703	ENSTGUG00000001403	XM_002198497.2	ENSTGUT00000001465	XP_002198533.1	ENSTGUP00000001452	yes	Full	
Takifugu rubripes	tru	Torafugu	Cry1	cryptochrome 1 (CRY1)	CAAB02001844.1	ENSTRUG00000014032	--	ENSTRUT00000036025	--	ENSTRUP00000035895	yes	Full	
			Cry2	cryptochrome-2-like; cryptochrome 2 (CRY2)	101061376	ENSTRUG00000015322	XM_003976633.1	ENSTRUT00000039292	XP_003976682.1	ENSTRUP00000039151	no	Full	
			Cry3	cryptochrome 2a (CRY2a)	CAAB02010204-08	ENSTRUG00000001852	--	ENSTRUT00000004295	--	ENSTRUP00000004271	no	partial	
			Cry5	cryptochrome 5	101062553	ENSTRUG00000003780	XM_003969514.1	ENSTRUT00000008943	XP_003969563.1	ENSTRUP00000008892	no	Full	
Tetranychus urticae	tur	Two-spotted spider mite	Cry1	--	CAEY01002034.1	tetur09g05920	--	tetur09g05920.1	--	tetur09g05920.1	no	partial	
			Cry6	--	CAEY01000279.1	tetur16g02770	--	tetur16g02770.1	--	tetur16g02770.1	no	Full	
Trichuris trichiura	ttr	Human whipworm	no crys										
Varroa destructor	vde	Varroa mite	no crys										
Volvox carteri	vca	Spherical alga	CryA1	DNA photolyase	9621417		XM_002945880.1		XP_002945926.1		no	partial	
Xenopus tropicalis	xtr	Western clawed frog	Cry1	cryptochrome 1 (photolyase-like) CRY1	550065	ENSXETG00000016951	NM_001017311.3	ENSXETT00000062200	NP_001017311.2	ENSXETP00000059100	yes/no	Full	
			Cry2	cryptochrome 2 (photolyase-like) CRY2	100422810	ENSXETG00000001023	XM_002934092.2	ENSXETT00000002232	XP_002934138.2	ENSXETP00000002232	no	Full	
			Cry4	cryptochrome 4 (cry4); Cry1 protein	100170456	ENSXETG00000015354	NM_001130234.1	ENSXETT00000033570	NP_001123706.1	ENSXETP00000033570	yes/no	Full	
			Cry5	--	100144974	ENSXETG00000003913	NM_001126540.1	ENSXETT00000008500	NP_001120012.1	ENSXETP00000008500	no/yes	Full	
Zootermopsis nevadensis	zne	Nevada Dampwood Termite	Cry1										
Zea mays	zma	Corn	CryP1	--	100280217	GRMZM2G052821	NM_001153148.1	GRMZM2G052821_T01	NP_001146620.1	GRMZM2G052821_P01	no/yes	Full	
