# Supplementary material for: Eumetazoan Cryptochrome Phylogeny and Evolution
Source: Genome Biol Evol. 2015 Jan 18;7(2):601–19. doi: 10.1093/gbe/evv010 (PMC4350181; doi:10.1093/gbe/evv010)
Supplement: Supplementary Data [file supp_evv010_New_Microsoft_Office_Word_Document.docx]

**Supplementary Files**

Supplementary table 1: Table with Latin names, common names and accession numbers.
